# CD155/SRC complex promotes hepatocellular carcinoma progression via inhibiting the p38 MAPK signalling pathway and correlates with poor prognosis

**DOI:** 10.1002/ctm2.794

**Published:** 2022-04-05

**Authors:** An‐Li Jin, Chun‐Yan Zhang, Wen‐Jing Zheng, Jing‐Rong Xian, Wen‐Jing Yang, Te Liu, Wei Chen, Tong Li, Bei‐Li Wang, Bai‐Shen Pan, Qian Li, Jian‐Wen Cheng, Peng‐Xiang Wang, Bo Hu, Jian Zhou, Jia Fan, Xin‐Rong Yang, Wei Guo

**Affiliations:** ^1^ Department of Laboratory Medicine Zhongshan Hospital Fudan University Shanghai P. R. China; ^2^ Department of Liver Surgery & Transplantation Liver Cancer Institute Zhongshan Hospital Fudan University Key Laboratory of Carcinogenesis and Cancer Invasion Ministry of Education Shanghai P. R. China; ^3^ Department of Hepatobiliary Surgery Shenzhen Key Laboratory Guangdong Provincial Key Laboratory of Regional Immunity and Diseases International Cancer Center Shenzhen University General Hospital Shenzhen University Clinical Medical Academy Shenzhen University Shenzhen Guangdong P.R. China; ^4^ Cancer Center Zhongshan Hospital Fudan University Shanghai P. R. China; ^5^ Department of Laboratory Medicine Xiamen Branch Zhongshan Hospital Fudan University Xiamen P. R. China; ^6^ Department of Laboratory Medicine Wusong Branch Zhongshan Hospital Fudan University Shanghai P. R. China; ^7^ Shanghai Geriatric Institute of Chinese Medicine Shanghai University of Traditional Chinese Medicine Shanghai P. R. China

**Keywords:** epithelial–mesenchymal transition, hepatocellular carcinoma, poliovirus receptor, prognosis, SRC

## Abstract

**Background:**

Hepatocellular carcinoma (HCC) is a prevalent malignancy with poor prognosis. As a cell adhesion molecule, poliovirus receptor (PVR/CD155) is abnormally overexpressed in tumour cells, and related to tumour proliferation and invasion. However, the potential role and mechanism of CD155 have not yet been elucidated in HCC.

**Methods:**

Immunohistochemistry, RT‐PCR and Western blot assays were used to determine CD155 expression in HCC cell lines and tissues. Cell Counting Kit‐8 and colony formation assays were used to examine cell proliferation. Transwell and wound healing assays were used to evaluate cell migration and invasion. Cell apoptosis and cycle distribution were assessed by flow cytometry. Cox regression and Kaplan–Meier analyses were performed to explore the clinical significance of CD155. The role of CD155 in vivo was evaluated by establishing liver orthotropic xenograft mice model. RNA sequencing, bioinformatics analysis and co‐immunoprecipitation assay were used to explore the downstream signalling pathway of CD155.

**Results:**

CD155 was upregulated in HCC tissues and represented a promising prognostic indicator for HCC patients (*n* = 189) undergoing curative resection. High CD155 expression enhanced cell proliferation, migration and invasion, and contributed to cell survival in HCC. CD155 overexpression also induced epithelial–mesenchymal transition in HCC cells. CD155 function in HCC involved SRC/p38 MAPK signalling pathway. CD155 interacted with SRC homology‐2 domain of SRC and promoted SRC activation, further inhibiting the downstream p38 MAPK signalling pathway in HCC.

**Conclusions:**

CD155 promotes HCC progression via the SRC/p38 MAPK signalling pathway. CD155 may represent a predictor for poor postsurgery prognosis in HCC patients.

## Graphical abstract

- CD155 is highly expressed in hepatocellular carcinoma (HCC) tissues, and its overexpression predicts poor postsurgery prognosis in HCC patients.
- CD155 promotes HCC progression via its cell‐intrinsic role in regulating cell proliferation, migration, invasion and epithelial–mesenchymal transition (EMT).
- CD155 enhances SRC activation through interacting with SH2 domain of SRC, further inhibiting the downstream p38 MAPK signalling pathway in HCC.

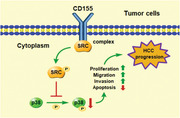

## 1 BACKGROUND

Hepatocellular carcinoma (HCC) is the most prevalent type of primary liver cancer that has become the sixth most commonly diagnosed cancer and the third leading cause of cancer death in 2020, with approximately 906 000 new cases and 830 000 deaths.^1^ Curative resection remains the most effective way for HCC treatment, but high rates of postoperative recurrence and metastasis have limited the efficacy of surgery.^2^ As the underlying mechanism of HCC progression remains enigmatic, it is critical to explore the underlying mechanism associated with recurrence and metastasis of HCC to identify new potential therapeutic targets.

Poliovirus receptor (PVR/CD155) was initially identified as a receptor that mediated poliovirus infection.^3^ Subsequent researches reported that CD155 played an important role in regulating cell adhesion, migration, polarisation and proliferation.^4^, ^5^ Recent studies have shown that CD155 is abnormally overexpressed in human cancers, and its aberrant expression is associated with the malignant phenotypes of cancers.^6^ High CD155 expression predicted poor prognosis in liver hepatocellular carcinoma (LIHC) cohort from The Cancer Genome Atlas (TCGA) database. Although the role of CD155 in tumour progression is gradually revealed, its function and regulatory mechanism are still poorly understood in HCC.

SRC is a non‐receptor tyrosine kinase, and its protein expression and kinase activity are frequently augmented in many human cancers.^7^ It had been reported that SRC could regulate cell adhesion, invasion, proliferation and apoptosis to promote the metastatic phenotype of HCC.^8^ Previous studies showed that CD155 recruited SRC homology‐2 domain‐containing tyrosine phosphatase‐2 (SHP‐2) to initiate intracellular signal transduction, which contained the same SRC homology‐2 (SH2) domain as SRC.^9^, ^10^ In addition, CD155 was shown to enhance glioma cell migration and dispersal via SRC/focal adhesion kinase signalling.^11^ These findings implied a potential connection between CD155 and SRC. Nevertheless, the regulation of CD155 and SRC and the crosstalk between them in HCC have not been elucidated.

In this research, we examined the potential function of CD155 in HCC cells. Both in vitro and in vivo results showed that high CD155 expression contributed to tumour growth and metastasis in HCC. RNA sequencing (RNA‐seq), bioinformatics analysis, co‐immunoprecipitation (co‐IP) assay and in vitro experiments revealed that CD155 interacted with SRC and enhanced SRC activity via CD155/SRC complex formation, subsequently inhibiting p38 MAPK signalling pathway in HCC progression. The prognostic value of CD155 in HCC patients had also been explored. Our data indicated that targeting CD155 might be a strategy to reduce HCC progression and metastasis, thus having important therapeutic implications for HCC patients.

## 2 METHODS

### 2.1 Patients and follow‐up

Two groups of HCC patients were enrolled in our research. For tissue microarrays (TMAs) analysis, 189 HCC patients (group I) undergoing curative resection in the Liver Cancer Institute, Zhongshan Hospital, Fudan University from 2012 to 2013 were enrolled, and paired peritumoural and tumour tissues of each patient were collected. In 2019–2020, another HCC cohort (*n* = 20, group II) was enrolled to analyse the infiltration of immune cells in HCC tissues from patients undergoing curative resection. Tumour stage was determined using China liver cancer (CNLC) staging system.^12^ Complete clinical information of each patient was available. Overall survival (OS) was defined as the time from the date of curative resection to death. Time to recurrence (TTR) was defined as the time between the curative resection and the first time of discovering intrahepatic recurrence or extrahepatic metastasis. Survival data were collected until June 2018. Informed consent was obtained from each patient enrolled in our study.

### 2.2 Statistics

SPSS software 16.0 and Graphpad prism 8.0 were used for statistical analysis. The significance of differences in data between groups was analysed by chi‐square test, Fisher's exact test, Student's *t*‐test and Mann–Whitney *U* test. OS and TTR were assessed by Kaplan–Meier survival curves and log‐rank test. Univariate and multivariate analyses of prognostic parameters were performed by establishing Cox proportional hazards models. *p*‐Value <.05 was considered statistically significant.

## 3 RESULTS

### 3.1 High CD155 expression predicts worse prognosis in HCC

**FIGURE 1:**
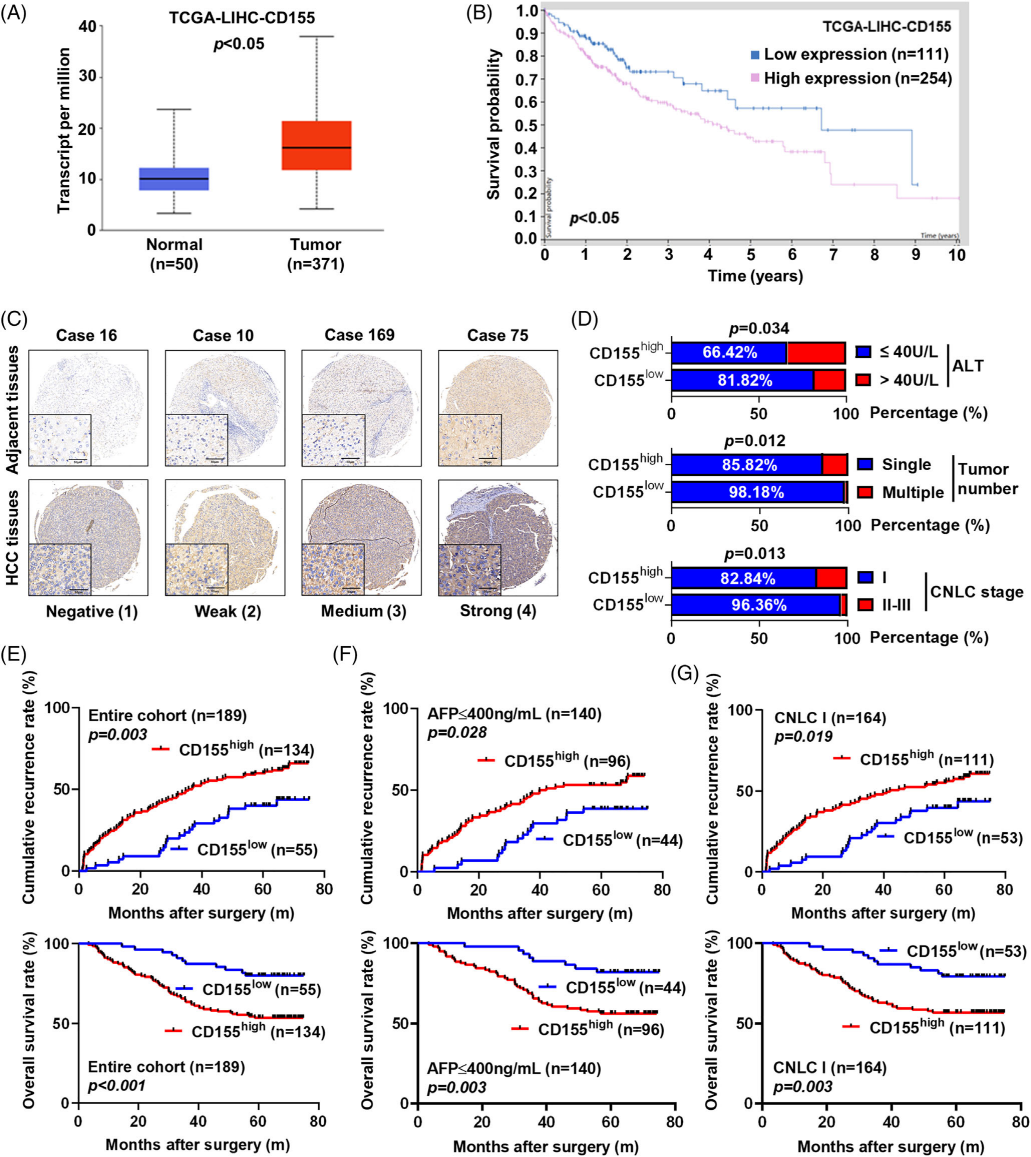
CD155 represents a prognostic biomarker in hepatocellular carcinoma (HCC). (A) The analysis of differential expression of CD155 in The Cancer Genome Atlas‐liver hepatocellular carcinoma (TCGA‐LIHC) was performed by the UALCAN database. (B) The analysis of overall survival (OS) of CD155 in TCGA‐LIHC was performed by the Human Protein Atlas database. (C) CD155 was highly expressed in tumour tissues compared with adjacent tissues. Representative immunohistochemistry (IHC) staining images of CD155 expression in tumour tissues and adjacent tissues in tissue microarrays (TMAs) (*n* = 189) are shown. Scale bar: 50 μm. (D) Correlation between CD155 expression and clinicopathological characteristics of HCC patients in TMAs (*n* = 189); chi‐square tests and Fisher's exact tests were used. (E) Kaplan–Meier analysis of OS and time to recurrence (TTR) of 189 HCC patients according to CD155 expression level; log‐rank tests were used. (F and G) Kaplan–Meier analysis of OS and TTR of HCC patients in low recurrent risk subgroups (AFP ≤400 ng/ml and CNLC stage I); log‐rank tests were used

CD155 was highly expressed in HCC tissues and cell lines, and high CD155 expression correlated with poor prognosis in TCGA‐LIHC cohort (Figure 1A,B; Figure S1A,B). We further investigated CD155 protein expression in TMAs containing specimens from 189 HCC patients who underwent curative resection. We found that CD155 expression in HCC tissues was higher than that in adjacent tissues (Figure 1C). According to the results of immunohistochemistry (IHC) staining, 134 HCC patients were classified as having high CD155 expression and 55 HCC patients were considered as having low CD155 expression. High CD155 expression was associated with high alanine aminotransferase (ALT) level (*p *= .034), multiple tumours (*p *= .012) and advanced CNLC stage (*p *= .013) (Figure 1D; Table 1). Kaplan–Meier analysis showed that CD155^high^ patients had a significantly shorter OS (*p *< .001) and TTR (*p *= .003) than CD155^low^ patients (Figure 1E). Univariate analysis showed that ALT, aspartate transaminase (AST), α‐fetoprotein (AFP), number of tumours, tumour size, vascular invasion, Edmondson stage, CNLC stage and CD155 expression (HR = 1.98, 95% CI: 1.25–3.14, *p *= .004) were associated with TTR of HCC patients, while vascular invasion and CD155 expression (HR = 2.92, 95% CI: 1.54–5.55, *p *= .001) were related to OS of HCC patients (Table 2; Figure S2A,B). Notably, multivariate analysis indicated that high CD155 expression was an independent indicator for predicting both TTR (HR = 1.83, 95% CI: 1.13–2.97, *p *= .014) and OS (HR = 2.87, 95% CI: 1.51–5.45, *p *= .001) in HCC patients (Table 3; Figure S2A,B).

**TABLE 1:** Correlation between CD155 expression and clinicopathological characteristics of HCC patients in group I

		CD155 expression		
Variables	*N*	Low (55)	High (134)	*p*
Sex				
Male	151	40	111	.115
Female	38	15	23	
Age, years				
>50	110	33	77	.748
≤50	79	22	57	
Child Pugh score				
A	179	53	126	.769
B	10	2	8	
Liver cirrhosis				
No	42	17	25	.066
Yes	147	38	109	
ALT, U/L				
≤40	134	45	89	**.034**
>40	55	10	45	
AST, U/L				
≤40	135	39	96	.919
>40	54	16	38	
AFP, ng/ml				
≤400	140	44	96	.234
>400	49	11	38	
Number of tumours				
Single	169	54	115	**.012**
Multiple	20	1	19	
Tumour size, cm				
≤5	119	36	83	.650
>5	70	19	51	
Tumour encapsulation				
Complete	123	37	86	.685
None	66	18	48	
Satellite lesion				
No	171	51	120	.499
Yes	18	4	14	
Vascular invasion				
No	107	33	74	.547
Yes	82	22	60	
Edmondson stage				
I–II	126	37	89	.910
III–IV	63	18	45	
CNLC stage				
I	164	53	111	**.013**
II–III	25	2	23	

Abbreviations: AFP, α‐fetoprotein; ALT, alanine aminotransferase; AST, aspartate transaminase; CNLC, China liver cancer; HCC, hepatocellular carcinoma.

**TABLE 2:** Univariate Cox proportional regression analysis of factors associated with recurrence and overall survival in HCC

	Recurrence		Overall survival	
Variables	HR (95% CI)	*p*	HR (95% CI)	*p*
Age (>50 vs. ≤50 years)	0.73 (0.50–1.07)	.104	0.64 (0.41–1.02)	.060
Liver cirrhosis (yes vs. no)	1.52 (0.92–2.49)	.099	1.11 (0.63–1.96)	.712
ALT (>40 vs. ≤40 U/L)	1.57 (1.06–2.34)	**.025**	1.80 (0.85–2.24)	.190
AST (>40 vs. ≤40 U/L)	1.78 (1.20–2.64)	**.004**	1.49 (0.92–2.41)	.101
AFP (>400 vs. ≤400 ng/ml)	1.86 (1.25–2.78)	**.002**	1.49 (0.91–2.43)	.117
Number of tumours (multiple vs. single)	1.81 (1.06–3.08)	**.030**	1.57 (0.84–3.02)	.157
Tumour size (>5 vs. ≤5 cm)	2.35 (1.60–3.43)	**<.001**	1.59 (0.99–2.48)	.056
Tumour encapsulation (none vs. complete)	1.15 (0.78–1.69)	.452	1.03 (0.64–1.67)	.903
Satellite lesions (yes vs. no)	1.67 (0.96–2.88)	.069	0.78 (0.33–1.76)	.528
Vascular invasion (yes vs. no)	2.08 (1.42–3.04)	**<.001**	1.91 (1.21–3.04)	**.006**
Edmondson stage (III–IV vs. I–II)	1.78 (1.21–2.61)	**.003**	1.50 (0.94–2.39)	.087
CNLC stage (II–III vs. I)	1.89 (1.17–3.05)	**.009**	1.58 (0.88–2.84)	.122
CD155 (high vs. low)	1.98 (1.25–3.14)	**.004**	2.92 (1.54–5.55)	**.001**

Abbreviations: AFP, α‐fetoprotein; ALT, alanine aminotransferase; AST, aspartate transaminase; CI, confidence interval; CNLC, China liver cancer; HR, hazard ratio.

**TABLE 3:** Multivariate Cox proportional regression analysis of factors associated with recurrence and overall survival in HCC

	Recurrence		Overall survival	
Variables	HR (95% CI)	*p*	HR (95% CI)	*p*
ALT (>40 vs. ≤40 U/L)	1.13 (0.72–1.77)	.595	N.A.	N.A.
AST (>40 vs. ≤40 U/L)	1.38 (0.87–2.20)	.168	N.A.	N.A.
AFP (>400 vs. ≤400 ng/ml)	1.67 (1.09–2.55)	**.018**	N.A.	N.A.
Number of tumours (multiple vs. single)	1.47 (0.73–2.95)	.285	N.A.	N.A.
Tumour size (>5 vs. ≤5 cm)	1.62 (1.02–2.56)	**.041**	N.A.	N.A.
Vascular invasion (yes vs. no)	1.38 (0.87–2.18)	.175	1.87 (1.18–2.97)	**.008**
Edmondson stage (III–IV vs. I–II)	1.72 (1.14–2.57)	**.009**	N.A.	N.A.
CNLC stage (II–III vs. I)	0.78 (0.40–1.54)	.477	N.A.	N.A.
CD155 (high vs. low)	1.83 (1.13–2.97)	**.014**	2.87 (1.51–5.45)	**.001**

Abbreviations: AFP, α‐fetoprotein; ALT, alanine aminotransferase; AST, aspartate transaminase; CI, confidence interval; CNLC, China liver cancer; HR, hazard ratio; N.A., not applicable.

The prognostic value of CD155 in clinical subgroups with low recurrent risks (AFP ≤400 ng/ml; tumour size ≤5 cm; single‐tumour lesion; Edmondson stages I–II; CNLC stage I)^13^ had also been investigated in HCC, and we found that the prognostic significance of CD155 remained in these subgroups (Figure 1F,G; Figure S3A–C). In the AFP ≤400 ng/ml group, the 5‐year TTR rate of CD155^high^ patients was 46.9% compared with 61.4% for CD155^low^ patients (*p *= .028). The rates for CD155^high^ patients and CD155^low^ patients were 44.9% and 60.4% in the CNLC stage I group (*p *= .019), 50.4% and 75.0% in the tumour size ≤5 cm group (*p *= .006), 43.5% and 61.1% in the single‐tumour lesion group (*p *= .009) and 45.9% and 70.3% in the Edmondson stages I–II group (*p *= .004), respectively (Figure 1F,G; Figure S3A–C).

### 3.2 CD155 promotes aggressive phenotypes of HCC cells in vitro

**FIGURE 2:**
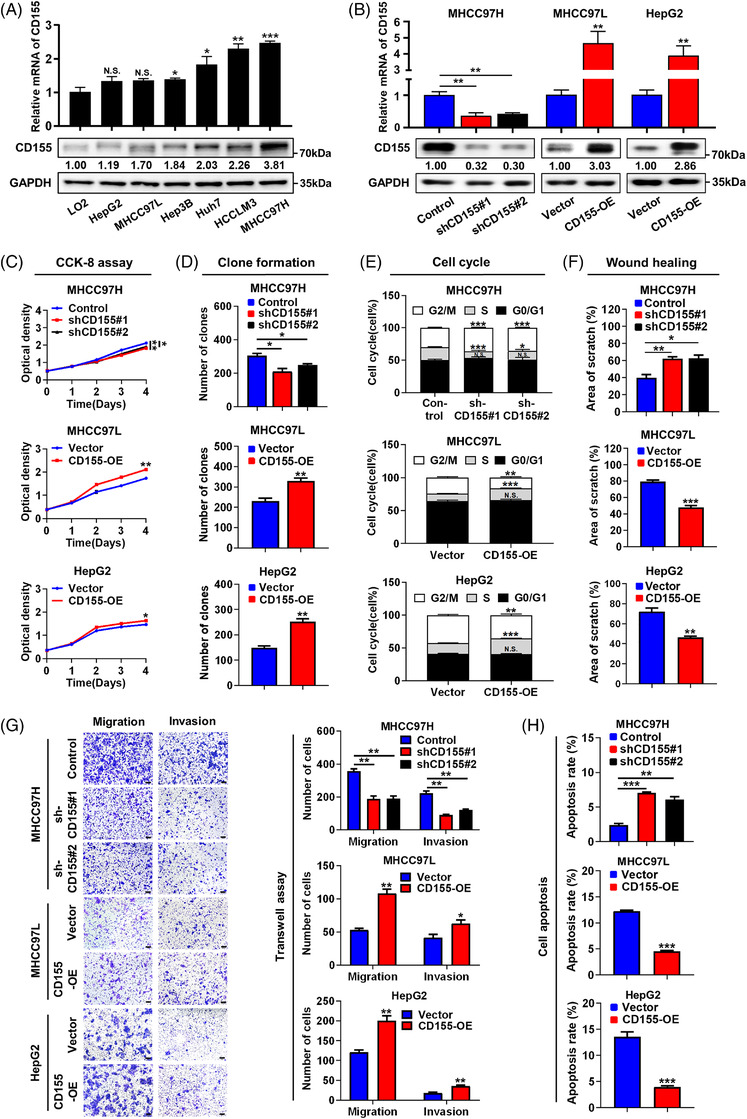
CD155 promotes proliferation, migration and invasion, and reduces apoptosis of hepatocellular carcinoma (HCC) cells in vitro. (A) mRNA and protein expression levels of CD155 in HCC cell lines were detected by RT‐PCR and Western blot (WB) assays; *t*‐tests were used. (B) Efficiencies of CD155 knockdown and overexpression were validated by RT‐PCR and WB assays; *t*‐tests were used. (C and D) Influence of CD155 on HCC cell proliferation was evaluated by Cell Counting Kit‐8 and colony formation assays; *t*‐tests were used. (E) Influence of CD155 on HCC cell cycle was evaluated by flow cytometry; *t*‐tests were used. (F and G) Influence of CD155 on HCC cell migration and invasion was evaluated by wound healing and transwell assays; *t*‐tests were used. Scale bar: 100 μm. (H) Influence of CD155 on HCC cell apoptosis was evaluated by flow cytometry; *t*‐tests were used. Error bars represent the standard error of mean (SEM) from at least three independent experiments. *p < .05; **p < .01; ***p < .001

The mRNA and protein expression levels of CD155 in HCC cell lines were determined by RT‐PCR and Western blot (WB) assays. HCC cells with higher metastatic potential^14^ (such as MHCC97H, HCCLM3, etc.) expressed higher CD155 expression than tumour cells with lower metastatic potential (such as MHCC97L, HepG2, etc.) (Figure 2A). To examine the role of CD155 in HCC cells, short hairpin RNA (shRNA) was used to decrease CD155 expression in MHCC97H cells, whereas an overexpression plasmid was used to stably overexpress CD155 in MHCC97L and HepG2 cells. Our results showed that CD155 was successfully knocked down and overexpressed, respectively (Figure 2B). In vitro experiments revealed that CD155 knockdown inhibited cell growth in HCC, whereas CD155 overexpression promoted cell proliferation (Figure 2C,D; Figure S4A). Moreover, CD155 knockdown induced G2/M arrest in HCC cells, whereas CD155 overexpression had the opposite effects (Figure 2E; Figure S4B). Transwell and wound healing assays showed that CD155 knockdown suppressed cell migration and invasion, whereas CD155 overexpression accelerated these activities (Figure 2F,G; Figure S4C). Apoptosis assay indicated that CD155 knockdown led to a higher apoptosis rate of HCC cells, whereas CD155 overexpression contributed to cell survival (Figure 2H; Figure S4D).

### 3.3 CD155 induces EMT in HCC

**FIGURE 3:**
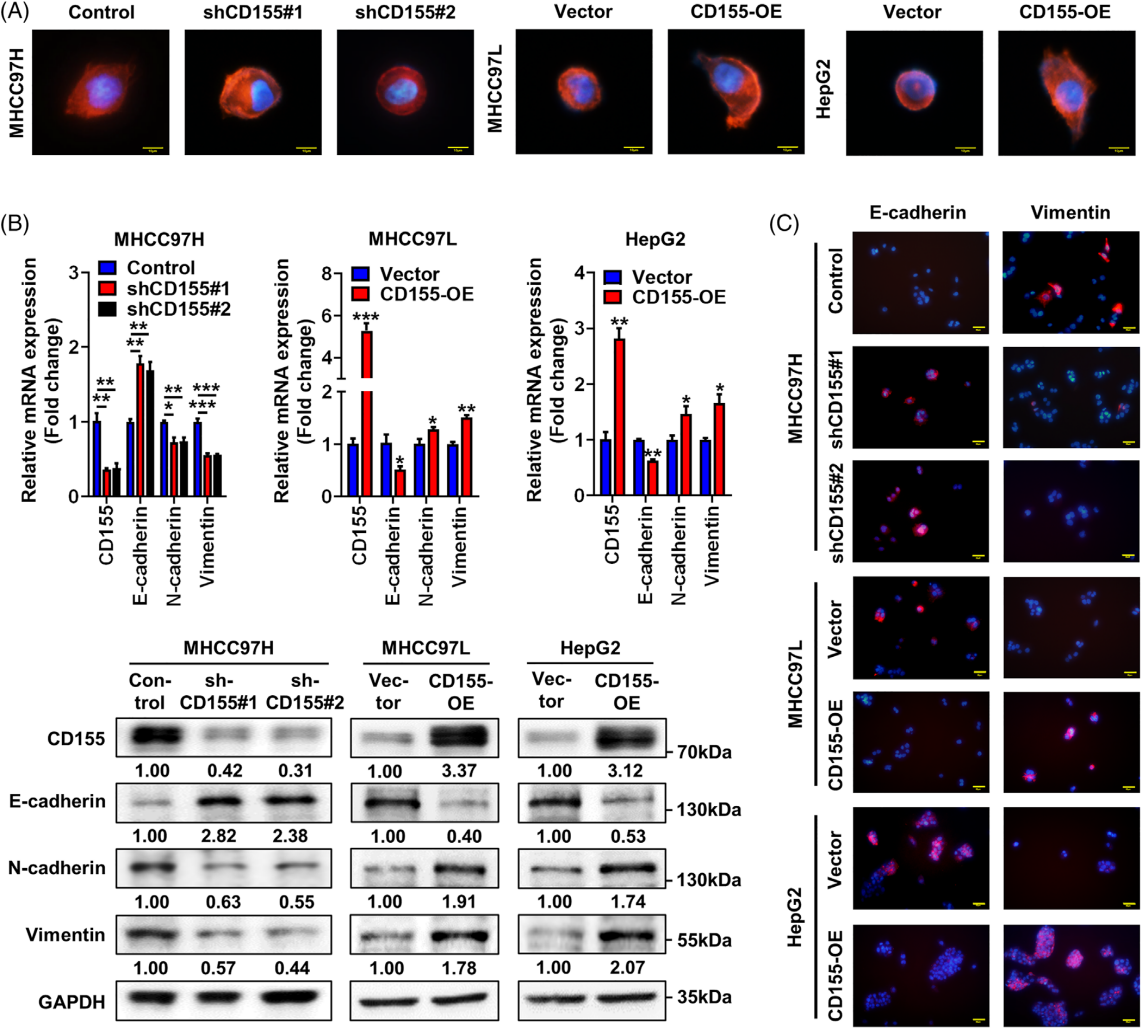
CD155 triggers epithelial–mesenchymal transition (EMT) in hepatocellular carcinoma (HCC) cells. (A) Representative phalloidin staining images of CD155 knockdown and overexpression HCC cells. Scale bar: 10 μm. (B) mRNA and protein expression levels of EMT‐related markers in CD155 knockdown and overexpression HCC cells were detected by RT‐PCR and Western blot (WB) assays; *t*‐tests were used. (C) Representative immunofluorescence staining images of EMT‐related markers in CD155 knockdown and overexpression HCC cells. Scale bar: 50 μm. Error bars represent the SEM from at least three independent experiments. *p < .05; **p < .01; ***p < .001

Epithelial–mesenchymal transition (EMT) is the significant feature of primary tumour formation and metastasis.^15^ We found that CD155 promoted cell invasion in HCC, and therefore we next investigated whether CD155 participated in EMT in HCC. The results demonstrated that CD155 knockdown cells showed an epithelial cobblestone phenotype with fewer pseudopods, whereas CD155 overexpression cells exhibited a spindle‐like cell shape with more pseudopods (Figure 3A). The expression levels of EMT‐related markers were determined by RT‐PCR and WB assays. CD155 knockdown resulted in higher E‐cadherin expression and lower expression levels of N‐cadherin and vimentin, whereas CD155 overexpression had the opposite effects (Figure 3B). Immunofluorescence assay also indicated that high CD155 expression correlated with lower E‐cadherin expression and higher vimentin expression in HCC cells (Figure 3C).

### 3.4 CD155 promotes HCC growth and metastasis in vivo

**FIGURE 4:**
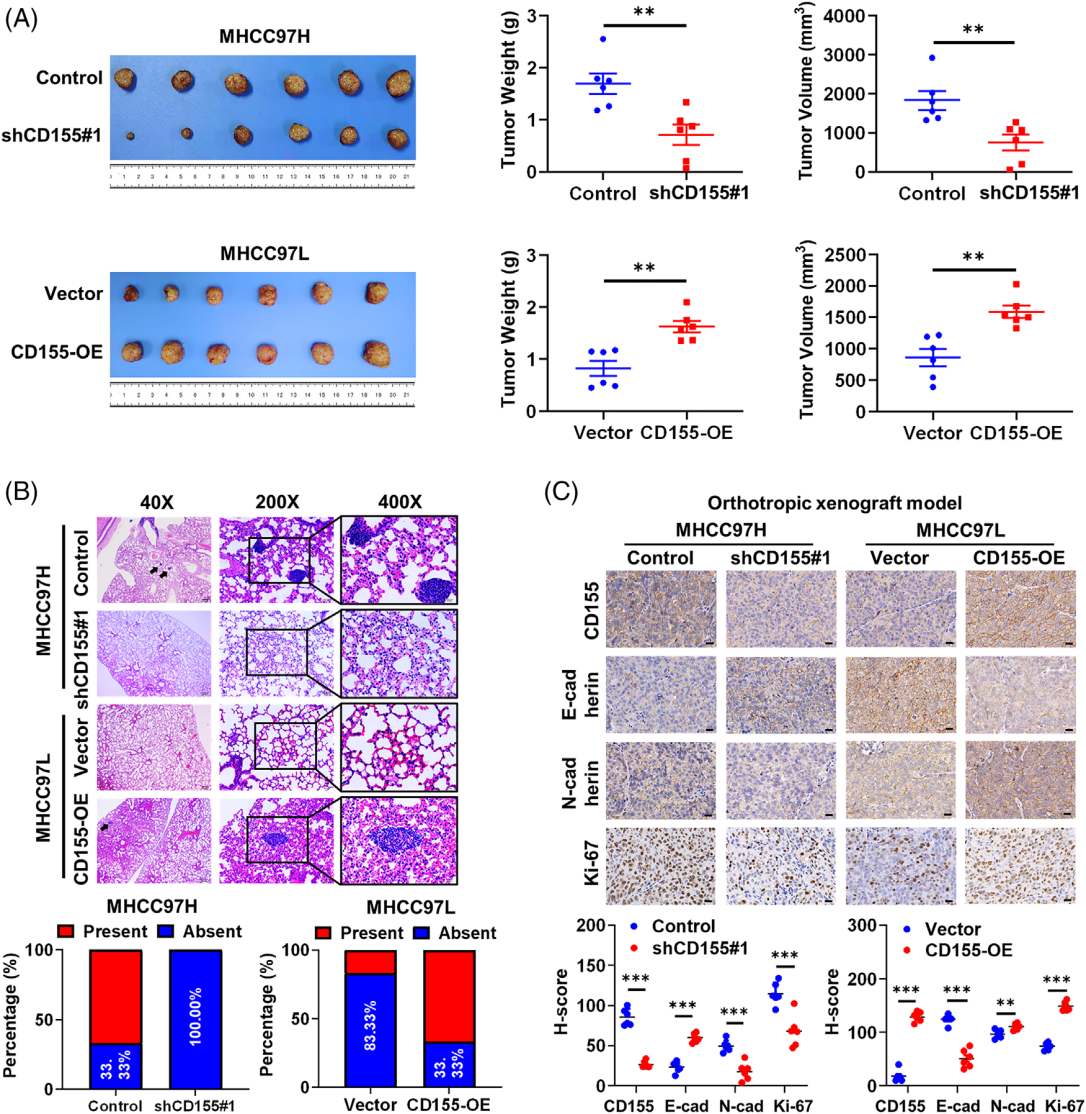
CD155 promotes growth and metastasis of hepatocellular carcinoma (HCC) cells in vivo. (A) Establishment of the orthotropic xenograft model with BALB/c‐nu mice (*n* = 6 in each group); *t*‐tests were used. (B) Representative haematoxylin–eosin (HE) staining images of lung metastasis in orthotropic xenograft mice model. (C) Representative immunohistochemistry (IHC) staining images of CD155, E‐cadherin, N‐cadherin and Ki‐67 in tumour tissues from orthotropic xenograft mice model; *t*‐tests were used. Scale bar: 20 μm. Error bars represent the SEM from at least three independent experiments. *p < .05; **p < .01; ***p < .001

Based on our in vitro results, we next assessed the role of CD155 in vivo via establishing the liver orthotropic xenograft mice model using MHCC97H and MHCC97L cells. The results showed that CD155 knockdown inhibited tumour growth, whereas CD155 overexpression had the opposite effects (Figure 4A). We also evaluated the presence of lung metastasis by haematoxylin–eosin (HE) staining. We found that high CD155 expression led to a higher incidence rate of lung metastasis compared with low CD155 expression group (Figure 4B). IHC staining assay also showed that high CD155 expression in tumours was related to lower E‐cadherin expression and higher expression levels of N‐cadherin and Ki‐67 (Figure 4C).

### 3.5 CD155 promotes HCC progression via the p38 MAPK signalling pathway

**FIGURE 5:**
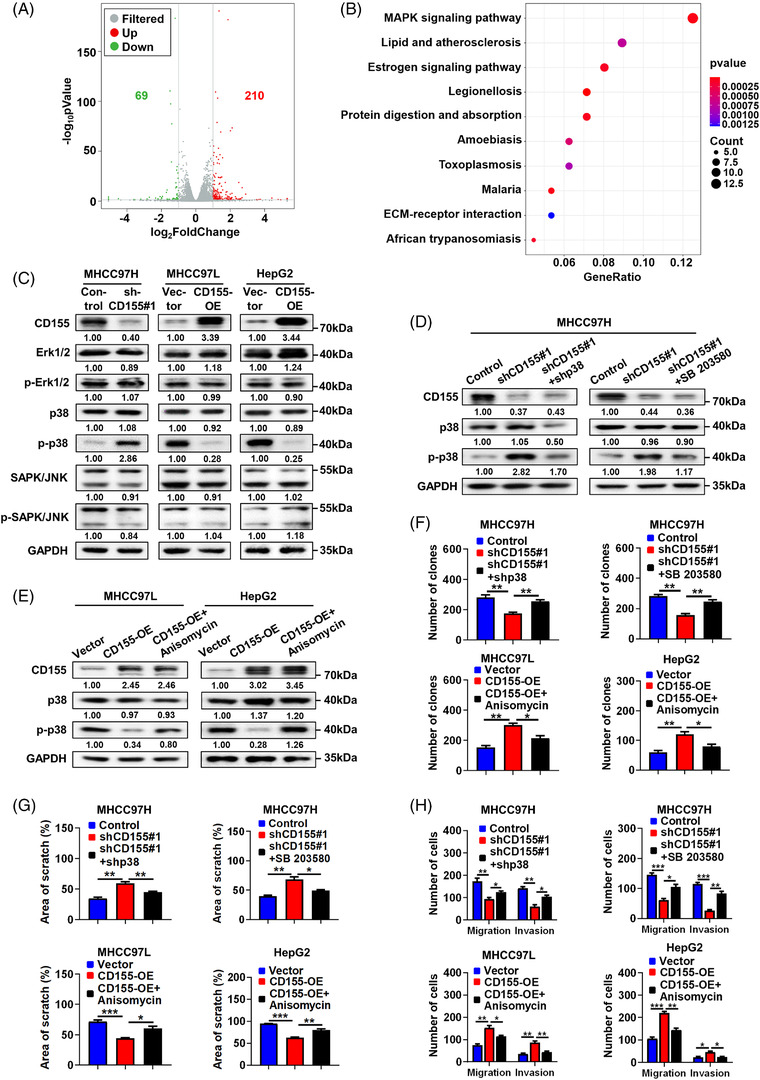
The p38 MAPK signalling pathway is the downstream signalling pathway of CD155 in hepatocellular carcinoma (HCC). (A) RNA‐seq was used for DEGs analysis and volcano plot of DEGs is shown (*n* = 3 in each group). (B) Top 10 enrichment KEGG pathways of DEGs. (C) Expressions of Erk1/2, p38, SAPK/JNK and their phosphorylated forms in CD155 knockdown and overexpression HCC cells were detected by Western blot (WB) assay. (D and E) Expressions of p38 and p‐p38 in CD155 knockdown HCC cells transfected with shp38 or treated with SB 203580 (10 μM for 30 min) and CD155 overexpression HCC cells treated with anisomycin (5 μM for 30 min) were detected by WB assay. (F) Proliferation ability of indicated HCC cells was evaluated by colony formation assay; *t*‐tests were used. (G and H) Migration and invasion abilities of indicated HCC cells were evaluated by wound healing and transwell assays; *t*‐tests were used. Error bars represent the SEM from at least three independent experiments. *p < .05; **p < .01; ***p < .001

To further explore the downstream signalling pathway of CD155 in HCC, we used RNA‐seq to identify differentially expressed genes (DEGs) between CD155 knockdown MHCC97H cells and control MHCC97H cells (*n* = 3 in each group), and 210 upregulated genes and 69 downregulated genes were identified in CD155 knockdown cells (*p *< .05, |log_2_FoldChange| > 1) (Figure 5A). By performing GO analysis of DEGs, genes related to biological process, cellular component and molecular function with details are shown in Figure S5A. The top 10 KEGG pathways of DEGs were explored, and the MAPK signalling pathway was the top enriched pathway (Figure 5B).

To evaluate whether CD155 regulated the MAPK signalling pathway, we detected expression levels of Erk1/2, p38, SAPK/JNK and their phosphorylated forms using WB assay. The results showed that CD155 knockdown in HCC cells resulted in increased p38 phosphorylation, whereas CD155 overexpression led to decreased p38 phosphorylation; and the level of total p38 remained unchanged (Figure 5C). We did not detect any significant change in the phosphorylation levels of Erk1/2 and SAPK/JNK (Figure 5C).

We next explored whether CD155 exerted its effects in HCC through the p38 MAPK signalling pathway. SB 203580 (a p38 MAPK inhibitor) and shRNA were used to inhibit p38 MAPK signalling, whereas anisomycin (a p38 MAPK activator) was used to activate p38 MAPK signalling (Figure 5D,E). In vitro experiments demonstrated that inhibition of p38 MAPK signalling enhanced cell proliferation, migration and invasion in CD155 knockdown cells, whereas activation of p38 MAPK signalling decreased these activities in CD155 overexpression cells (Figure 5F–H; Figure S5B–D).

### 3.6 CD155 interacting with SRC contributes to inhibiting the p38 MAPK signalling pathway

**FIGURE 6:**
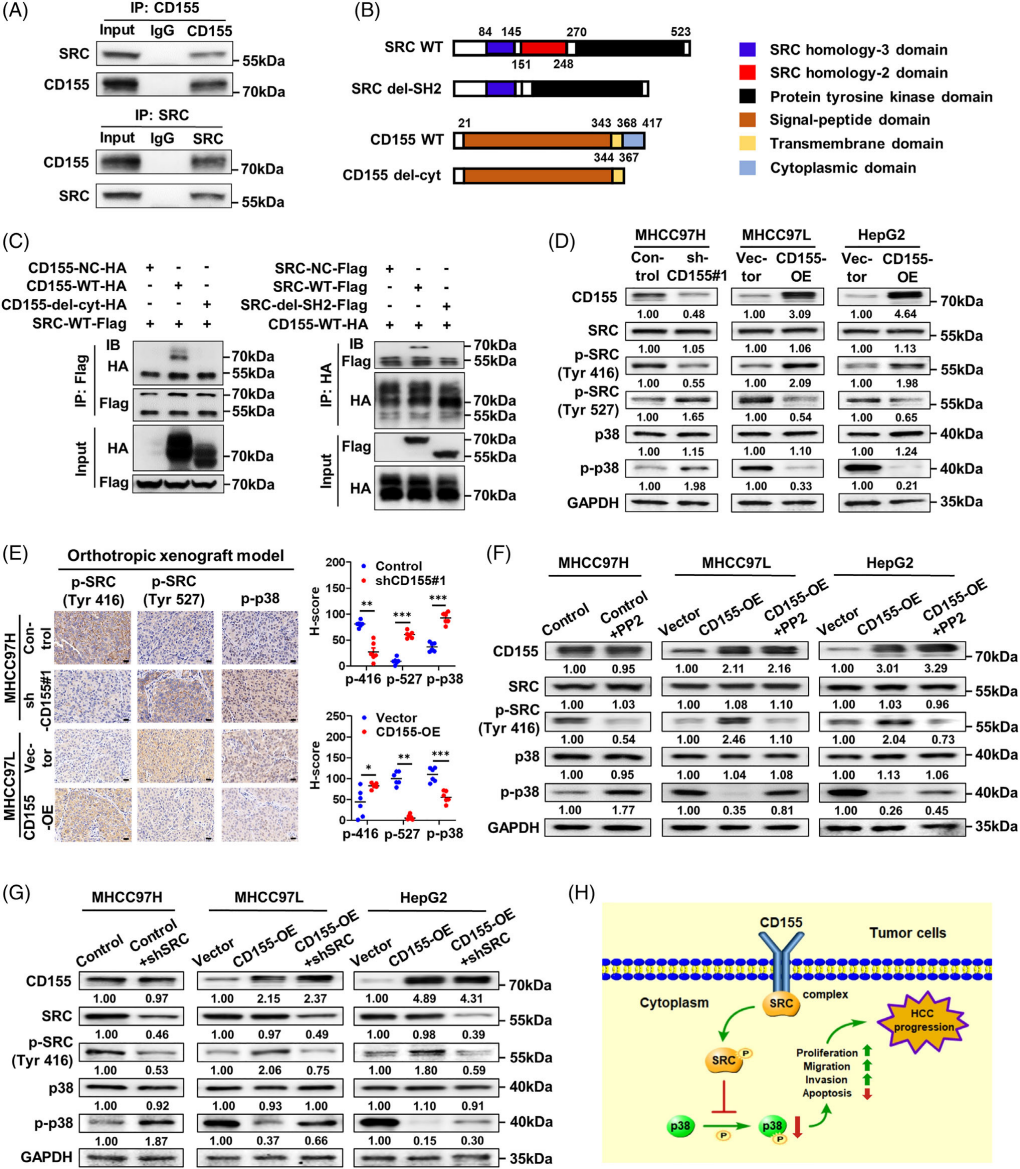
CD155/SRC complex formation inhibits the p38 MAPK signalling pathway in hepatocellular carcinoma (HCC). (A) The interaction between CD155 and SRC was detected by co‐IP assay in MHCC97H cells. (B) Diagram of SRC WT, SRC del‐SH2, CD155 WT and CD155 del‐cyt. (C) The binding domains of CD155 and SRC were identified by co‐IP assay in HEK293T cells. (D) Expressions of SRC, p38 and their phosphorylated forms in CD155 knockdown and overexpression HCC cells were detected by Western blot (WB) assay. (E) Representative immunohistochemistry (IHC) staining images of p‐SRC (Tyr 416), p‐SRC (Tyr 527) and p‐p38 in tumour tissues from orthotropic xenograft mice model; *t*‐tests and Mann–Whitney *U* tests were used. Scale bar: 20 μm. (F and G) Expressions of SRC, p38 and their phosphorylated forms in high CD155 expression HCC cells treated with PP2 (20 μM for 24 h) or transfected with shSRC were detected by WB assay. (H) A schematic diagram showing underlying molecular mechanism of CD155 in HCC. Error bars represent the SEM from at least three independent experiments. *p < .05; **p < .01; ***p < .001

We predicted potential interacting proteins of CD155 by STRING database to explore the mechanism by which CD155 inhibited the p38 MAPK signalling pathway in HCC. We identified 11 potential CD155‐interacting proteins with the highest confidence, including CD226, CD96, SRC, etc. (Table S1; Figure S6A). Previous studies showed that CD155 interacted with SHP‐2, which contained the same SH2 domain as SRC,^9^ implying that there was a potential binding site between CD155 and SRC. This interaction was further confirmed by co‐IP assay (Figure 6A). Subsequently, we investigated which domains of CD155 and SRC mediated the interaction between them. SH2 domain is an important domain that regulates the activation of SRC.^16^ We conducted the truncations with a C‐terminal Flag tag as SRC wild‐type (WT)‐Flag and SRC del‐SH2‐Flag, and a C‐terminal HA tag as CD155 WT‐HA and CD155 del‐cyt‐HA (Figure 6B). Our results demonstrated that cytoplasmic domain of CD155 and SH2 domain of SRC contributed to the interaction between them (Figure 6C).

We next examined the functional consequence of CD155 interaction with SRC. WB assay showed that CD155 knockdown inhibited SRC phosphorylation at Tyr 416 (a phosphorylation site that promotes SRC activation) and promoted SRC phosphorylation at Tyr 527 (a phosphorylation site that inhibits SRC activation); CD155 overexpression had the opposite effects (Figure 6D). Moreover, IHC staining based on orthotropic xenograft mice model demonstrated that high CD155 expression was related to higher p‐SRC (Tyr 416) expression and lower expression levels of p‐SRC (Tyr 527) and p‐p38 (Figure 6E).

We further explored whether CD155‐mediated effects in HCC were dependent on SRC activation. PP2 (an SRC inhibitor) and shRNA were used to inhibit SRC signalling in high CD155 expression cells. We found that phosphorylation of p38 was enhanced after inhibition of SRC signalling (Figure 6F,G). In high CD155 expression cells, cell proliferation, migration and invasion were inhibited when cells were treated with PP2 or transfected with shSRC (Figure S6B–D).

## 4 DISCUSSION

Postoperative tumour recurrence and metastasis are major factors that limit the survival of HCC patients undergoing curative resection.^2^ Elucidating underlying mechanisms in the occurrence and development of HCC is critical to explore novel strategies to reduce recurrence and metastasis and further improve the efficacy of surgery. In this research, we found that cells with high CD155 expression correlated with higher proliferative and metastatic potential. CD155 was overexpressed in HCC tissues and predicted poor prognosis. Both in vitro and in vivo results indicated that high CD155 expression contributed to HCC growth and metastasis. Further investigations revealed that CD155 interacted with SRC and enhanced SRC activation, then inhibiting the downstream p38 MAPK signalling pathway to promote HCC progression. These findings indicated that CD155 might serve as a pro‐tumorigenic molecule in HCC and provided evidences for the importance of CD155/SRC/p38 MAPK signalling pathway in HCC progression.

CD155 was reported to be expressed at low levels on immune cells,^17^ endothelial cells^18^ and tumour‐infiltrating myeloid cells.^19^ In addition, CD155 was highly expressed in tumour cells of multiple cancer types and predicted poor prognosis, such as breast cancer,^20^ pancreatic cancer,^21^ cholangiocarcinoma,^22^ etc. Several researches have been done to explore cell‐extrinsic biology of CD155 acting as an immunomodulatory molecule in tumour progression,^6^, ^23^ but little is known about cell‐intrinsic biology of CD155 in HCC cells, including its involvement in cell–cell adhesion, motility and proliferation.^4^, ^5^ Our study emphasised on the cell‐intrinsic role of CD155 in HCC and revealed its pro‐tumorigenic role. Mechanistically, our study proposed that CD155/SRC complex formation inhibited p38 phosphorylation and contributed to the aggressive phenotype of HCC cells. p38 MAPK contains four subtypes, which are encoded by different genes, including p38α (MAPK14), p38β (MAPK11), p38γ (MAPK12) and p38δ (MAPK13).^24^ p38α is widely expressed in most cell types at high levels, whereas p38β, p38γ and p38δ are restrictedly expressed at low levels.^25^ Some studies have illustrated that p38α functions as an important tumour suppressor in tumour progression, while others also identified the pro‐tumorigenic role of p38α.^26^ Previous studies demonstrated that mice with liver‐specific deletion of p38α showed enhanced tumour growth and development.^27^, ^28^ p38α also reduced the accumulation of reactive oxygen species and further inhibited fibrogenesis and consequent tumour progression in HCC.^29^ In our study, we found that knockdown of CD155 promoted p38 phosphorylation in HCC cells, and both p38 MAPK inhibitor and shRNA targeting p38α in CD155 knockdown cells resulted in enhanced cell growth and aggressiveness, which revealed a potential tumour‐suppressor role of p38 MAPK as the downstream molecule of CD155 in HCC. Subsequently, we explored an interaction between cytoplasmic domain of CD155 and SH2 domain of SRC. SRC mainly comprise SH3 domain, SH2 domain and protein tyrosine kinase domain.^30^ Inactivation of SRC occurs when its Tyr 527 is phosphorylated and it binds back to the SH2 domain, and this interaction results in a closed molecular structure without the potential for autophosphorylation.^31^ In our study, we proposed that the interaction between CD155 and SRC resulted in augmented SRC activation by disrupting the intramolecular interaction that held SRC in a close molecular structure, representing a new mechanism for the abnormal activation of SRC in HCC. Our results also showed that both SRC inhibitor and shSRC increased p38 phosphorylation in high CD155 expression cells in HCC, which were in accordance with previous studies.^32^, ^33^ A possible hypothesis for the regulation of p38 activation by SRC is that SRC activation may inhibit events occurring upstream of p38 phosphorylation in a CD155‐dependent manner in HCC. Due to critical cell‐extrinsic role of CD155, we also analysed immune cell infiltration in HCC tissues to explore the correlation between tumour immune microenvironment states and CD155 expression. We found that numbers of CD8^+^ T cells and CD56^+^ NK cells were decreased in HCC tissues from CD155^high^ patients (*n* = 10) compared with CD155^low^ patients (*n* = 10) (Figure S7A,B). Moreover, our results of TMAs demonstrated that high CD155 expression was related to high ALT level, indicating liver damage and inflammation,^34^ which might be related to the role of CD155 as an immunomodulatory molecule.

The prognostic significance of CD155 was also investigated in this research. In our HCC cohort, CD155^high^ patients (134/189) had a shorter TTR and OS compared with CD155^low^ patients, which was in accordance with the previous study in HCC.^35^ However, the prognostic significance of CD155 in low recurrent risk subgroups of HCC has not been explored, which may be more useful to yield specific findings. AFP has long been used as an effective biomarker for HCC diagnosis, and high serum level of AFP usually indicates the occurrence and progression of HCC.^36^ There has been a lack of biomarkers to predict prognosis in AFP‐negative HCC patients.^37^ When we stratified the HCC patients by AFP level, the results demonstrated that prognostic value of CD155 remained in AFP ≤400 ng/ml subgroup (5‐year TTR rate CD155^high^ vs. CD155^low^: 46.9% vs. 61.4%, *p *= .028). Compared with other HCC staging systems, CNLC staging system is more suitable for Chinese patients with a hepatitis B virus‐positive background, and CNLC stage I is usually associated with successful surgical resection and better prognosis of HCC patients.^12^ The prognostic potential of CD155 in CNLC stage I subgroup had also been explored, and we found that CD155^high^ patients had a shorter TTR and OS compared with CD155^low^ patients (5‐year TTR rate CD155^high^ vs. CD155^low^: 44.9% vs. 60.4%, *p *= .019). The clinical significance of CD155 in these low recurrent risk subgroups enabled clinicians to identify HCC patients with a high risk of metastasis and recurrence and implement adjuvant therapies after curative resection.

A previous study reported that HCC patients with higher CD155 expression had a longer OS, which was contrary to our results^38^; one possible reason for this discrepancy was the difference in the characteristics of enrolled population. More than half (57.3%) of the patients in their study were diagnosed with poor‐differentiation HCC, whereas most (66.7%) of HCC patients in our research were diagnosed as Edmondson stages I–II. Another report also showed that loss of CD155 expression predicted poor prognosis in HCC^39^; the discrepancy might be attributed to a difference in the criteria of CD155 expression in Kaplan–Meier analysis. Only percentage of positive cells was used to stratify their HCC cohort, whereas the product of intensity and percentage scores was used in our study.

This study has several limitations. Previous studies have found that EMT has consequences for T‐cell dysfunction and T‐cell exclusion, which induced tumour escape.^40^, ^41^ Recent findings revealed that CD155 could interact with TIGIT/CD96 on CD8^+^ T cells and subsequently contributed to T‐cell dysfunction.^42^, ^43^ In addition, T cells could also be excluded from the tumour site by some immunosuppressive factors in tumour microenvironment.^44^ In this study, we found that high CD155 expression promoted EMT in HCC cells. Moreover, our results also indicated that numbers of CD3^+^, CD8^+^ and CD56^+^ cells were decreased in HCC tissues from CD155^high^ patients. However, the relationship between CD155 expression and T‐cell exclusion was not investigated in this study. Whether the EMT induced by CD155 would result in T‐cell dysfunction and T‐cell exclusion is not clear. These need further investigations for clarification.

## 5 CONCLUSIONS

Our study demonstrates that CD155 is overexpressed in HCC tissues and contributes to the aggressive phenotype of HCC cells. Mechanistically, SRC/p38 MAPK signalling pathway may be involved in CD155‐induced HCC progression. Overall, our study suggests that CD155 is expected to be a promising prognostic indicator and novel tumour immunotherapy target for HCC patients.

## CONFLICT OF INTEREST

The authors declare that there is no potential conflict of interest.

## Supporting information

Supporting Information

Supporting Information

Supporting Information

Supporting Information

Supporting Information

Supporting Information

Supporting Information

Supporting Information

Supporting Information

Supporting Information
