# Supplementary material for: CD155/SRC complex promotes hepatocellular carcinoma progression via inhibiting the p38 MAPK signalling pathway and correlates with poor prognosis
Source: Clin Transl Med. 2022 Apr 5;12(4):e794. doi: 10.1002/ctm2.794 (PMC8982318; doi:10.1002/ctm2.794)
Supplement: Supplementary file 1 — Supporting Information [file CTM2-12-e794-s008.docx]

**CD155/SRC complex promotes hepatocellular carcinoma progression via inhibiting the p38 MAPK signaling pathway and correlates with poor prognosis**

- **Supplementary figure legends**

**Supplementary figure 1: The expression of CD155 in public databases.** (A) The analysis of CD155 expression in human tumors was performed by the TIMER database. (B) The analysis of CD155 expression in tumor cell lines was performed by the Cancer Cell Line Encyclopedia database. *, *p* value<0.05; **, *p* value<0.01; ***, *p* value<0.001.

**Supplementary figure 2: Metanalysis plot of univariate and multivariate Cox proportional regression analysis of factors associated with TTR and OS.** (A) TTR. (B) OS.

**Supplementary figure 3: The prognostic significance of CD155 expression in low recurrent risk subgroups of HCC.** Kaplan-Meier analysis of OS and TTR of HCC patients in (A) tumor size≤5cm subgroup, (B) single tumor lesion subgroup and (C) Edmondson stage I-II subgroup; log-rank tests were used.

**Supplementary figure 4: The biological function of CD155 in HCC cell lines.** (A) Influence of CD155 on HCC cell proliferation was evaluated by colony formation assay. (B) Influence of CD155 on HCC cell cycle was evaluated by flow cytometry. (C) Influence of CD155 on HCC cell migration was evaluated by wound healing assay. Scale bar: 100μm. (D) Influence of CD155 on HCC cell apoptosis was evaluated by flow cytometry.

**Supplementary figure 5: The role of p38 MAPK signaling pathway in CD155-induced HCC progression.** (A) Most significantly enriched GO terms of DEGs were performed by R package. BP: biological process; CC: cellular component; MF: molecular function. (B) Proliferation ability of indicated HCC cells was evaluated by colony formation assay. (C-D) Migration and invasion abilities of indicated HCC cells were evaluated by wound healing and transwell assays. Scale bar: 100μm.

**Supplementary figure 6: CD155 promotes HCC progression via enhancing SRC activity.** (A) Protein-protein interaction network of CD155 with the highest confidence was performed by the STRING database. (B) Proliferation ability of indicated HCC cells was evaluated by colony formation assay; *t* tests were used. (C-D) Migration and invasion abilities of indicated HCC cells were evaluated by wound healing and transwell assays; *t* tests were used. Scale bar: 100μm. Error bars represent the SEM from at least three independent experiments. *, *p* value<0.05; **, *p* value<0.01; ***, *p* value<0.001.

**Supplementary figure 7:** **The correlation between CD155 expression and numbers of tumor-infiltrating lymphocytes in HCC tissues.** (A) Representative IHC staining images of CD3^+^, CD8^+^ and CD56^+^ cells in HCC tissues. Scale bar: 20μm. (B) Numbers of CD3^+^, CD8^+^ and CD56^+^ cells in HCC tissues from CD155^high^ (n=10) and CD155^low^ (n=10) patients, respectively; *t* tests and Mann-Whitney *U* tests were used. Error bars represent the SEM from at least three independent experiments. *, *p* value<0.05; **, *p* value<0.01; ***, *p* value<0.001.

- **Supplementary Tables**

**Supplementary Table S1. Prediction of proteins that potentially interact with CD155 according to STRING database.**

**Supplementary Table S2. Clinical characteristics of HCC patients in group II.**

**Supplementary Methods and Materials**

**Tissue microarrays (TMAs) analysis and immunohistochemistry (IHC) staining**

TMAs were constructed as previously reported (Outdo Biotech, Shanghai, China).^1^ IHC staining was performed as previously described.^2^ CD155 antibody (1:200, Cell Signaling Technology, CST, USA) was used for IHC staining of TMAs. The expression of CD155 was mainly detected on the tumor cell membrane and cytoplasm. Two investigators independently assessed the results of IHC staining. For the evaluation of CD155 expression, scores were assigned as intensity and percentage of positive staining of tumor cells, as follows: (1) Intensity: negative scored 1, weak scored 2, medium scored 3, and strong scored 4; (2) Proportion: <10% positive cells scored 0, 10%-40% positive cells scored 1, 40%-70% positive cells scored 2, and>70% positive cells scored 3. CD155 expression was determined using the following formula: overall scores = percentage score × intensity score. Overall scores of <8 and ≥8 were defined as low expression and high expression, respectively. For the evaluation of target molecule staining, histochemistry score (H-score) was used to assess the results of each sample. H-score = (percentage of weak intensity area × 1) + (percentage of moderate intensity area × 2) + (percentage of strong intensity area × 3).^3,4^ For cell count quantification, the mean value of the numbers of positive immune cells (cells/mm^2^) in tumor tissues was used as the quantification results of particular cell population.^5^ Other antibodies used for IHC staining included E-cadherin (1:200, CST), N-cadherin (1:100, CST), Ki-67 (1:200, CST), p-SRC (Tyr 416) (1:100, CST), p-SRC (Tyr 527) (1:50, CST), p-p38 (1:100, CST), CD3 (1:100, CST), CD8 (1:200, CST) and CD56 (1:200, Abcam, USA).

**Cell culture and transfection**

MHCC97H, HCCLM3, Huh7, Hep3B, MHCC97L, HepG2, LO2 and HEK293T cells were obtained from the Chinese Academy of Sciences (Shanghai, China). Cells were cultured in high-glucose Dulbecco’s modified Eagle’s medium (DMEM, Gibco BRL, USA) with 10% fetal bovine serum (FBS, Gibco BRL), 1% penicillin and 100μg/ml streptomycin (Gibco BRL) in a humidified incubator containing 5% CO_2_ at 37ºC. CD155 short hairpin RNA (shRNA) lentivirus (shCD155) and control lentivirus (Control) were constructed by Genechem (Shanghai, China). shCD155 oligos were cloned into GV493-Puro vector. The shRNA sequence for shCD155#1 was 5′-TTGCAGGTCACATTCTTGCCG-3′, for shCD155#2 was 5’-AATTGTTGTTGGCGTTTCGGG-3’, and for Control was 5′-TTCTCCGAACGTGTCACGT-3′. For CD155 overexpression, an expression plasmid GV492-CD155 was constructed by Genechem. Cells infected with lentivirus were selected in DMEM with 10% FBS and 1μg/ml of puromycin. The shRNA for SRC and p38 MAPK were conducted by Genechem. shSRC oligos and shp38 oligos were cloned into GV248-Puro vector. The shRNA sequence for shSRC was 5’-TTGCACACCAGGTTCTCTCCC-3’ and for shp38 was 5’-TTGGTAGATAAGGAACTGAAC-3’. For the protein truncation experiment, the plasmids, including SRC wild-type (WT), SRC del-SRC homology-2 (SH2) domain, CD155 WT and CD155 del-cytoplasmic (cyt) domain, were conducted by Genechem.

**RT-PCR**

Total RNA of cells was extracted by the RNA Isolation Kits (Qiagen, Germany), and cDNA was synthesized by the Quantitect Reverse Transcription Kit (Qiagen). All operations were carried out according to manufacturer’s instructions. The mRNA expression levels of genes were quantified by SYBR Mix (Takara, Japan) and Roche Real-time PCR Detection System (Roche Diagnostics). The primers used in this study were as follows: CD155: 5’-TGTCCACCTTCCAGCAGATG-3’(F), 5’-CCTCCTCCTCCAGCAGAATC-3’(R); E-cadherin: 5′-TACGCCTGGGACTCCACCTA-3′(F), 5′-CCAGAAACGGAGGCCTGAT-3′(R); N-cadherin: 5’-ATCCTACTGGACGGTTCG-3’(F), 5’-TTGGCTAATGGCACTTGA-3’(R); Vimentin: 5’-GAACGCCAGATGCGTGAAATG-3’(F), 5’-CCAGAGGGAGTGAATCCAGATTA-3’(R); GAPDH: 5′-GTCATCCAACGGGAATGCA-3′(F), 5′-TGATCGGTTACCGTGATCAAAA-3′(R). PCR conditions were as follows: 5 min at 95°C, followed by 40 cycles of 95°C for 10s and 60°C for 60s. GAPDH was used as an internal control in all PCR reactions. The fold change was calculated according to the formula 2^−ΔΔCt^. Each reaction was performed in triplicate.

**Western blot (WB)**

Cells were cultured in a 6-well plate with DMEM containing 10%FBS for 24 hours. For the addition of inhibitors, SB 203580 (10μM for 30 min), Anisomycin (5μM for 30 min) or PP2 (20μM for 24h) was added in the culture medium before cells were lysed. Cells were lysed in the RIPA buffer (Beyotime, Shanghai, China). It was placed at 4°C for 30 minutes and was shaken every 5 minutes. The supernatant was collected after centrifugation at 13,000×g for 20 minutes at 4°C. The BCA protein kit (Thermo Scientific, USA) was used to evaluate protein concentration. All lysates mixed with 5X sodium dodecyl sulfate-polyacrylamide gel electrophoresis (SDS-PAGE) sample loading buffer (Beyotime) were heated for 5 minutes at 100°C. Protein samples (20μg) were separated by SDS-PAGE, and transferred to a polyvinylidene fluoride (PVDF) membrane. First, the PVDF membrane was incubated with QuickBlock Blocking Buffer (Beyotime) for 30 minutes. Next, the membrane was incubated with primary antibody at 4°C for a night, followed by incubation with horseradish peroxidase-conjugated anti-rabbit/mouse antibody (Beyotime) for 2 hours. Primary antibodies were diluted in QuickBlock primary antibody dilution buffer (Beyotime) and antibodies dilutions were determined according to manufacturer’s instructions. Secondary antibodies were diluted in TBS (Sangon Biotech, Shanghai, China) containing 0.1% Tween-20 (Sangon) and antibodies dilutions were determined according to manufacturer’s instructions. The protein level was standardized to GAPDH, and then standardized to experimental control. Densitometric analysis of western blot was performed using NIH Image J software (Maryland, USA).

**Assays of cell proliferation, migration and invasion**

Cell Counting Kit-8 (CCK-8) assay and colony formation assay were used to determine cell proliferation. For CCK-8 assay, cells (5000 cells/100μl per well) were incubated in a 96-well plate. Cell viability were examined at 0 day, 1 day, 2 day, 3 day and 4 day. All operations were carried out according to manufacturer’s instructions. For colony formation assay, cells (1000 cells/2ml per well) were incubated in a 6-well plate with DMEM containing 10% FBS for 14 days. Every three days, cells were washed with PBS and cultured with DMEM containing 10% FBS. After 14 days, cells were fixed with 4% paraformaldehyde (Beyotime) and stained with Giemsa staining solution (Beyotime). Visible colonies were photographed and counted manually. Wound healing assay and transwell assay were used to evaluate cell migration and invasion. For wound healing assay, cells (5x10^4^ cells/500μl per well) were incubated in a 24-well plate for 24 hours. After entire petri dish was covered by cells, scratches were made on the cells using a sterile pipette tip to mimic the wound process. Next, cells were washed with PBS and cultured with DMEM containing 1% FBS. The images were photographed from the same field at 0 day and 2 day. For migration assay, cells (5x10^4^ cells/200μl per well) were incubated with DMEM without FBS in transwell chambers (Corning, USA). The lower chambers containing DMEM with 10% FBS attracted cells to migrate from the upper chambers. Cells were incubated at 37°C for 48 hours. Cells that had migrated to the lower surface of membrane were fixed with 4% paraformaldehyde and stained with Giemsa staining solution. Stained cells were counted under the microscope to assess the ability of cell migration. Except for treating the upper chamber with a MatriGel-coated membrane (dilution=1:8, Corning), the other steps of invasion assay were the same as the migration assay. All experiments were conducted in triplicate.

**Flow cytometry**

For apoptosis assay, cells were cultured in a 6-well plate with DMEM containing 10% FBS for 24 hours. APC Annexin V Apoptosis Detection Kit (BD, USA) was used according to the manufacturer’s instructions, and cell apoptosis was detected by Aria II flow cytometry (BD). For cell cycle assay, cells were synchronized with DMEM containing 0.1% FBS for 24 hours, and fixed with ice cold 70% ethanol for a night. Propidium iodide staining solution (BD) and RNase A (Beyotime) were used to treat fixed cells, and cell cycle was detected by Aria II flow cytometry (BD) and was analyzed by FlowJo software (BD). All experiments were conducted in triplicate.

**Antibodies and reagents**

These antibodies were used in this study: anti-CD155 (CST), anti-E-cadherin (CST), anti-N-cadherin (CST), anti-Vimentin (CST), anti-Erk1/2 (CST), anti-phospho-Erk1/2 (CST), anti-p38 (CST), anti-phospho-p38 (CST), anti-SAPK/JNK (CST), anti-phospho-SAPK/JNK (CST), anti-SRC (CST), anti-phospho-SRC (Tyr 416) (CST), anti-phospho-SRC (Tyr 527) (CST), anti-GAPDH (Beyotime), anti-Flag (CST) and anti-HA (CST). These reagents were used in this study: SB 203580 (MedChemExpress, MCE, Shanghai, China), Anisomycin (MCE) and PP2 (Selleck, Shanghai, China). For *in vitro* experiments, cells were cultured in a 6-well plate with DMEM containing 10%FBS for 24 hours. SB 203580 (10μM), Anisomycin (5μM) or PP2 (20μM) was added in the culture medium and co-cultured with cells for indicated time for further experiments.

**Immunofluorescence**

For cytoskeletal staining, cells were fixed with 4% paraformaldehyde and blocked with 5% bovine serum albumin. Next, cells were stained with 594-conjugated phalloidin (Abcam) for 60 minutes at 25°C, and cells were counterstained with DAPI (Beyotime) for observation under the microscope. For target molecule staining, cells were fixed with 4% paraformaldehyde and blocked with 5% bovine serum albumin. Next, cells were incubated with anti-E-cadherin (1:100, CST) and anti-Vimentin (1:100, CST) at 4°C for a night, followed by incubation with 594-conjugated anti-rabbit antibody (1:500, CST) for 2 hours, and cells were counterstained with DAPI for observation under the microscope.

**Co-IP**

Cells were lysed in the NP-40 buffer (Beyotime). Samples were slowly shaken and incubated with primary antibody at 4°C for a night. 40μl completely resuspended protein A+G agarose (Thermo Scientific) was added, and samples were slowly shaken at 4°C for 3 hours, followed by centrifugation at 2500 rpm for 5 minutes. The supernatant was carefully aspirated and washed with PBS for 5 times, and 40μl 1X SDS-PAGE loading buffer (Beyotime) was added to resuspend the pellet. Last, protein samples were heated for 5 minutes at 100°C, and were separated by SDS-PAGE. Anti-CD155 (Abcam, 1:30), anti-SRC (CST, 1:50), anti-Flag (CST, 1:50) and anti-HA (CST, 1:50) were used for co-IP assay.

***In vivo* assay**

To minimize the influence of the immune system in our study, BALB/c-nu mice purchased from the Department of Experimental Animals of the Chinese Academy of Sciences were used for experiments. The establishment of liver orthotropic xenograft mice model was conducted according to the previous study.^6^ First, 5×10^6^ cells were collected and resuspended in DMEM without FBS, and cells were injected subcutaneously into the left upper flank region of mice. After 6 weeks later, tumor tissues were removed and cut into pieces of the same size (1mm^3^). Next, we implanted it into the liver of each group respectively (n=6 in each group). At 8 weeks after implantation, mice were sacrificed and tumor tissues were obtained. Tumor volume was measured as [W(tumor width)^2^L(tumor length)]/2. For lung metastasis experiment, lung tissues from orthotropic xenograft mice model were harvested and fixed with 4% paraformaldehyde. Next, consecutive resection and haematoxylin-eosin (HE) staining were performed to evaluate the incidence of lung metastasis.

**RNA-sequencing (RNA-seq) and differentially expressed genes (DEGs) analysis**

Total RNA of cells was extracted by TRIzol reagent according to the manufacturer’s protocol. RNA purity and quantification were evaluated by NanoDrop 2000 spectrophotometer (Thermo Scientific). RNA integrity was evaluated by Agilent 2100 Bioanalyzer (Agilent Technologies, USA). The libraries were constructed using VAHTS Stranded mRNA-seq Library Prep Kit for Illumina V2 according to the manufacturer’s instructions. The transcriptome sequencing and analysis were performed by OE Biotech (Shanghai, China). The libraries were sequenced on an Illumina Novaseq 6000 platform and 150 bp paired-end reads were generated. Each sample (n=3 in each group) produced approximately 48.1M raw reads. First, we used Trimmomatic^7^ to process raw reads of fastq format, and removed low-quality reads to obtain clean reads. Then, about 46.8M clean reads for each sample were retained for subsequent analysis. We used HISAT2^8^ to map clean reads to the human genome (GRCh38). The FPKM^9^ of each gene was calculated by Cufflinks,^10^ and the read counts of each gene were obtained by HTSeq-count.^11^ Differential expression analysis was performed by the DESeq (2012) R package.^12^ *p*<0.05 and |log_2_FoldChange|>1 was set as the threshold for significantly differential expression. Hierarchical cluster analysis of DEGs was performed to demonstrate the expression of genes in different groups. Based on the hypergeometric distribution, GO enrichment and KEGG^13^ pathway enrichment analyses of DEGs were performed using R package, respectively.

**Bioinformatics analysis**

The analysis of the expression of CD155 in human tumors was performed by the TIMER database (<https://cistrome.shinyapps.io/timer/>).^14^ The analysis of the expression of CD155 in tumor cell lines was performed by the Cancer Cell Line Encyclopedia database (https://sites.broadinstitute.org/ccle).^15^ The analysis of the OS of CD155 in LIHC cohort from TCGA database was performed by the Human Protein Atlas database (<https://www.proteinatlas.org/>).^16^ The analysis of the differential expression of CD155 in TCGA-LIHC was performed by the UALCAN database (http://ualcan.path.uab.edu).^17^ Prediction of proteins that potentially interact with CD155 was performed by the STRING database (<https://www.string-db.org/>).^18^

**References**

1. Ma XL, Shen MN, Hu B, et al. CD73 promotes hepatocellular carcinoma progression and metastasis via activating PI3K/AKT signaling by inducing Rap1-mediated membrane localization of P110beta and predicts poor prognosis. *J HEMATOL ONCOL*. 2019;12(1):37.

2. Gao Q, Zhao YJ, Wang XY, et al. Activating mutations in PTPN3 promote cholangiocarcinoma cell proliferation and migration and are associated with tumor recurrence in patients. *GASTROENTEROLOGY*. 2014;146(5):1397-1407.

3. Maclean A, Bunni E, Makrydima S, et al. Fallopian tube epithelial cells express androgen receptor and have a distinct hormonal responsiveness when compared with endometrial epithelium. *HUM REPROD*. 2020;35(9):2097-2106.

4. Dogan S, Vasudevaraja V, Xu B, et al. DNA methylation-based classification of sinonasal undifferentiated carcinoma. *Mod Pathol*. 2019;32(10):1447-1459.

5. Benonisson H, Altintas I, Sluijter M, et al. CD3-Bispecific Antibody Therapy Turns Solid Tumors into Inflammatory Sites but Does Not Install Protective Memory. *MOL CANCER THER*. 2019;18(2):312-322.

6. Xiao S, Chang RM, Yang MY, et al. Actin-like 6A predicts poor prognosis of hepatocellular carcinoma and promotes metastasis and epithelial-mesenchymal transition. *HEPATOLOGY*. 2016;63(4):1256-1271.

7. Bolger AM, Lohse M, Usadel B. Trimmomatic: a flexible trimmer for Illumina sequence data. *BIOINFORMATICS*. 2014;30(15):2114-2120.

8. Kim D, Langmead B, Salzberg SL. HISAT: a fast spliced aligner with low memory requirements. *NAT METHODS*. 2015;12(4):357-360.

9. Roberts A, Trapnell C, Donaghey J, Rinn JL, Pachter L. Improving RNA-Seq expression estimates by correcting for fragment bias. *GENOME BIOL*. 2011;12(3):R22.

10. Trapnell C, Williams BA, Pertea G, et al. Transcript assembly and quantification by RNA-Seq reveals unannotated transcripts and isoform switching during cell differentiation. *NAT BIOTECHNOL*. 2010;28(5):511-515.

11. Anders S, Pyl PT, Huber W. HTSeq--a Python framework to work with high-throughput sequencing data. *BIOINFORMATICS*. 2015;31(2):166-169.

12. Anders S, Huber W. Differential expression analysis for sequence count data. *GENOME BIOL*. 2010;11(10):R106.

13. Kanehisa M, Araki M, Goto S, et al. KEGG for linking genomes to life and the environment. *NUCLEIC ACIDS RES*. 2008;36(Database issue):D480-D484.

14. Li T, Fu J, Zeng Z, et al. TIMER2.0 for analysis of tumor-infiltrating immune cells. *NUCLEIC ACIDS RES*. 2020;48(W1):W509-W514.

15. Ghandi M, Huang FW, Jane-Valbuena J, et al. Next-generation characterization of the Cancer Cell Line Encyclopedia. *NATURE*. 2019;569(7757):503-508.

16. Uhlen M, Fagerberg L, Hallstrom BM, et al. Proteomics. Tissue-based map of the human proteome. *SCIENCE*. 2015;347(6220):1260419.

17. Chandrashekar DS, Bashel B, Balasubramanya S, et al. UALCAN: A Portal for Facilitating Tumor Subgroup Gene Expression and Survival Analyses. *NEOPLASIA*. 2017;19(8):649-658.

18. Szklarczyk D, Gable AL, Lyon D, et al. STRING v11: protein-protein association networks with increased coverage, supporting functional discovery in genome-wide experimental datasets. *NUCLEIC ACIDS RES*. 2019;47(D1):D607-D613.
