# Supplementary material for: CD155/SRC complex promotes hepatocellular carcinoma progression via inhibiting the p38 MAPK signalling pathway and correlates with poor prognosis
Source: Clin Transl Med. 2022 Apr 5;12(4):e794. doi: 10.1002/ctm2.794 (PMC8982318; doi:10.1002/ctm2.794)
Supplement: Supplementary file 2 — Supporting Information [file CTM2-12-e794-s004.docx]

| **Supplementary Table S1. Prediction of proteins that potentially interact with CD155 according to STRING database.** | | | | | | | | | | |
| --- | --- | --- | --- | --- | --- | --- | --- | --- | --- | --- |
| node1 | node2 | Neighborhood on chromosome | Gene fusion | Phylogenetic co-occurrence | Homology | Co-expression | Experimentally determined interaction | Database annotated | Automated textmining | Combinedscore |
| PVR | CD226 | 0.000 | 0.000 | 0.000 | 0.000 | 0.000 | 0.884 | 0.800 | 0.990 | 0.999 |
| PVR | CD96 | 0.000 | 0.000 | 0.000 | 0.000 | 0.000 | 0.886 | 0.800 | 0.988 | 0.999 |
| PVR | TIGIT | 0.000 | 0.000 | 0.000 | 0.000 | 0.000 | 0.932 | 0.800 | 0.989 | 0.999 |
| PVR | MLLT4 | 0.000 | 0.000 | 0.000 | 0.000 | 0.054 | 0.056 | 0.900 | 0.759 | 0.975 |
| PVR | PVRL3 | 0.000 | 0.000 | 0.000 | 0.708 | 0.000 | 0.663 | 0.900 | 0.988 | 0.975 |
| PVR | DYNLT1 | 0.000 | 0.000 | 0.000 | 0.000 | 0.000 | 0.270 | 0.000 | 0.966 | 0.974 |
| PVR | PVRL1 | 0.000 | 0.000 | 0.000 | 0.768 | 0.000 | 0.658 | 0.900 | 0.890 | 0.971 |
| PVR | VTN | 0.000 | 0.000 | 0.000 | 0.000 | 0.000 | 0.270 | 0.000 | 0.920 | 0.939 |
| PVR | SRC | 0.000 | 0.000 | 0.000 | 0.000 | 0.065 | 0.000 | 0.900 | 0.280 | 0.926 |
| PVR | ITGAV | 0.000 | 0.000 | 0.000 | 0.000 | 0.076 | 0.000 | 0.900 | 0.115 | 0.911 |
| PVR | ITGB3 | 0.000 | 0.000 | 0.000 | 0.000 | 0.062 | 0.000 | 0.900 | 0.080 | 0.906 |
| Abbreviations: PVR, poliovirus receptor; TIGIT, T cell immunoreceptor with Ig and ITIM domains; MLLT4/AFDN, afadin, adherens junction formation factor; PVRL3/NECTIN3, nectin cell adhesion molecule 3; DYNLT1, dynein light chain Tctex-type 1; PVRL1/NECTIN1, nectin cell adhesion molecule 1; VTN, vitronectin; ITGAV, integrin subunit alpha V; ITGB3, integrin subunit beta 3. | | | | | | | | | | |
