# Supplementary material for: CD155/SRC complex promotes hepatocellular carcinoma progression via inhibiting the p38 MAPK signalling pathway and correlates with poor prognosis
Source: Clin Transl Med. 2022 Apr 5;12(4):e794. doi: 10.1002/ctm2.794 (PMC8982318; doi:10.1002/ctm2.794)
Supplement: Supplementary file 3 — Supporting Information [file CTM2-12-e794-s007.docx]

| **Supplementary Table S2. Clinical characteristics of HCC patients in group II.** | | | |
| --- | --- | --- | --- |
| Variables | N | CD155 expression | |
|  |  | Low (10) | High (10) |
| Sex | | | |
| Male | 16 | 8 | 8 |
| Female | 4 | 2 | 2 |
| Age, years | | | |
| >50 | 15 | 8 | 7 |
| ≤50 | 5 | 2 | 3 |
| Child-Pugh score | | | |
| A | 20 | 10 | 10 |
| B | 0 | 0 | 0 |
| Liver cirrhosis | | | |
| No | 13 | 7 | 6 |
| Yes | 7 | 3 | 4 |
| AFP, ng/mL | | | |
| ≤400 | 14 | 8 | 6 |
| >400 | 6 | 2 | 4 |
| Number of tumors | | | |
| Single | 13 | 5 | 8 |
| Multiple | 7 | 5 | 2 |
| Tumor size, cm | | | |
| ≤5 | 10 | 6 | 4 |
| >5 | 10 | 4 | 6 |
| Vascular invasion | | | |
| No | 3 | 1 | 2 |
| Yes | 17 | 9 | 8 |
| CNLC stage | | | |
| I | 10 | 5 | 5 |
| II-III | 10 | 5 | 5 |
| Abbreviations: AFP, α-fetoprotein; CNLC, China Liver Cancer. | | | |
