# Supplementary figures and images for: CD155/SRC complex promotes hepatocellular carcinoma progression via inhibiting the p38 MAPK signalling pathway and correlates with poor prognosis
Source: Clin Transl Med. 2022 Apr 5;12(4):e794. doi: 10.1002/ctm2.794 (PMC8982318; doi:10.1002/ctm2.794)

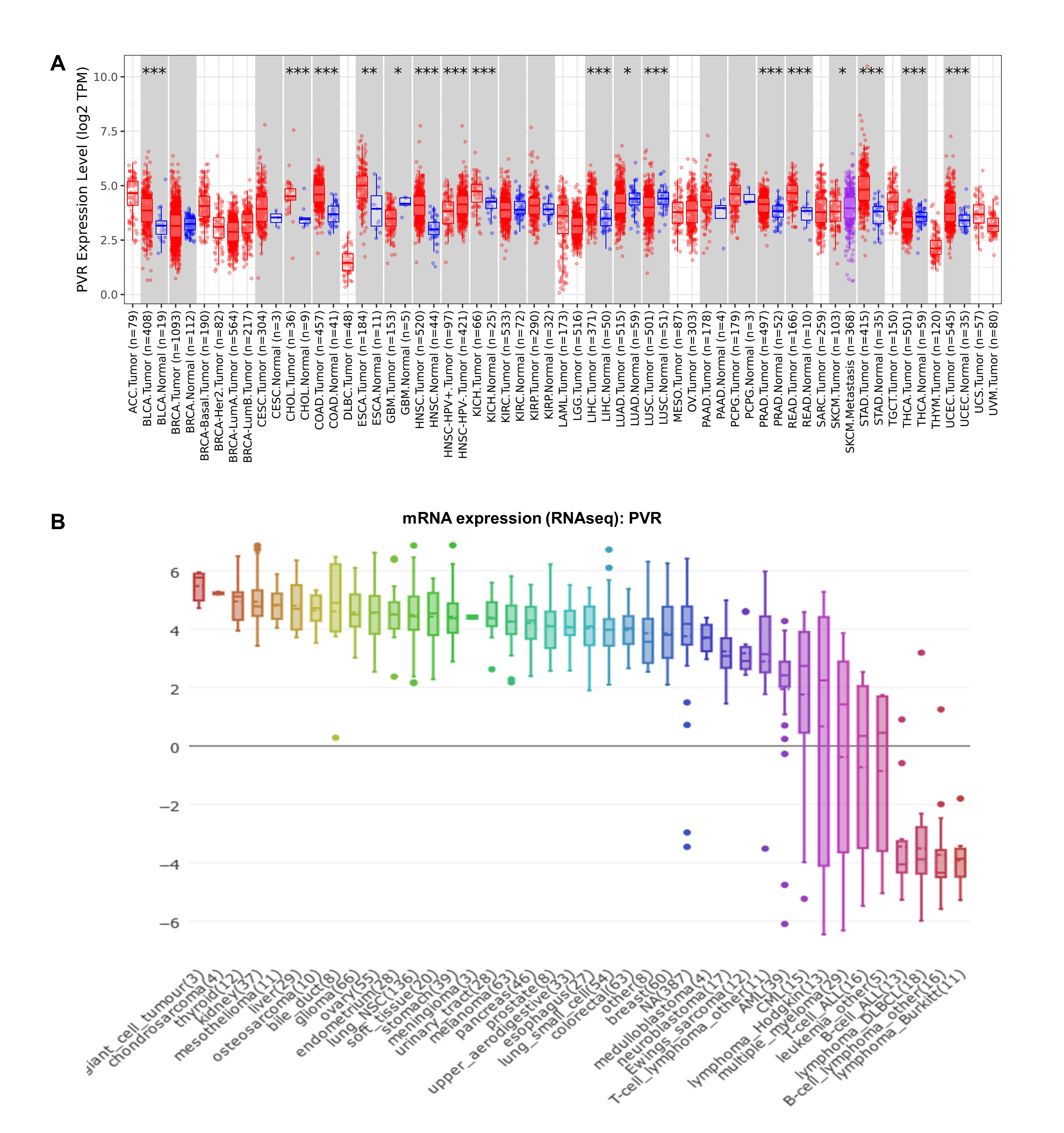

Supplement: Supplementary file 4 — Supporting Information [file CTM2-12-e794-s006.TIF]

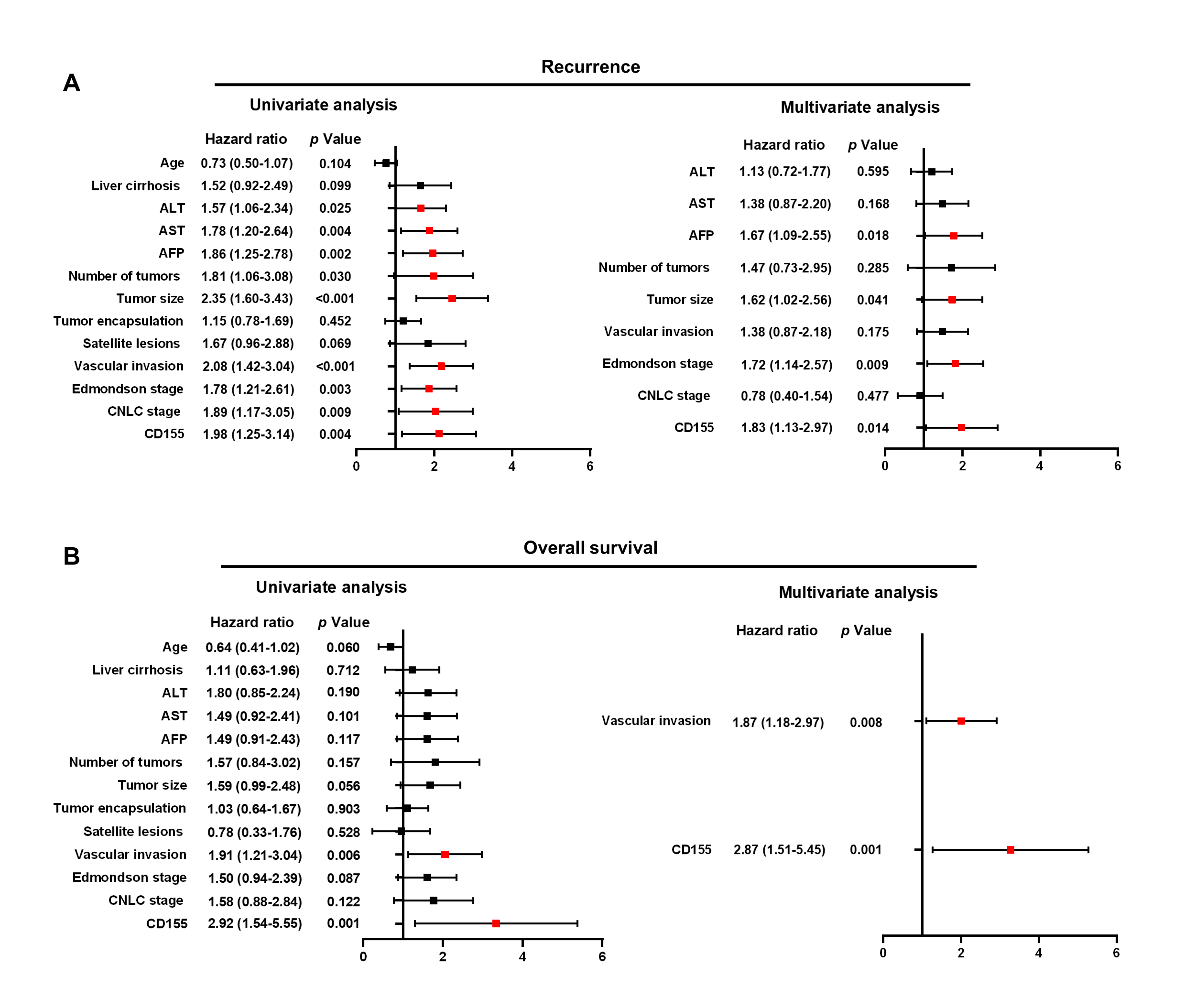

Supplement: Supplementary file 5 — Supporting Information [file CTM2-12-e794-s005.TIF]

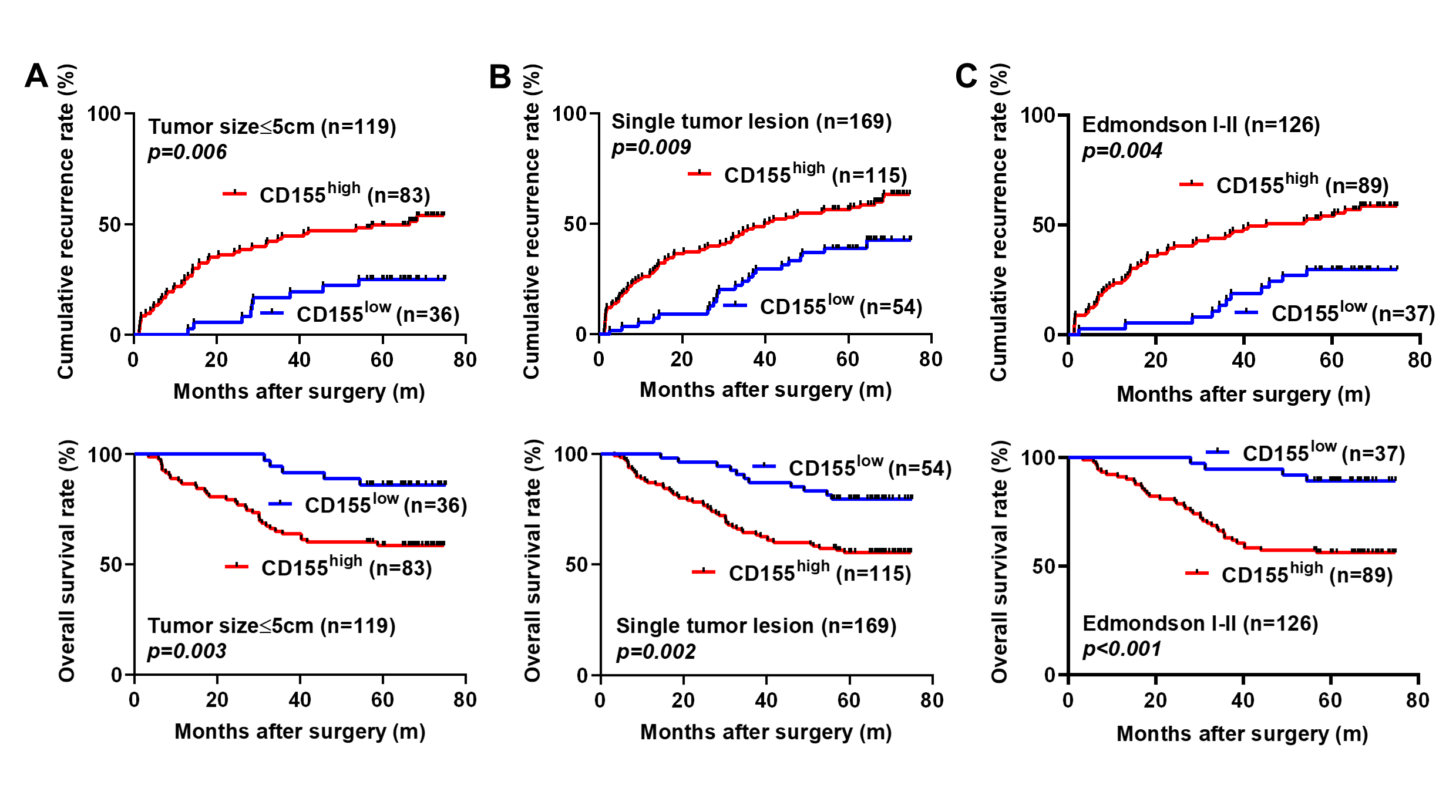

Supplement: Supplementary file 6 — Supporting Information [file CTM2-12-e794-s001.TIF]

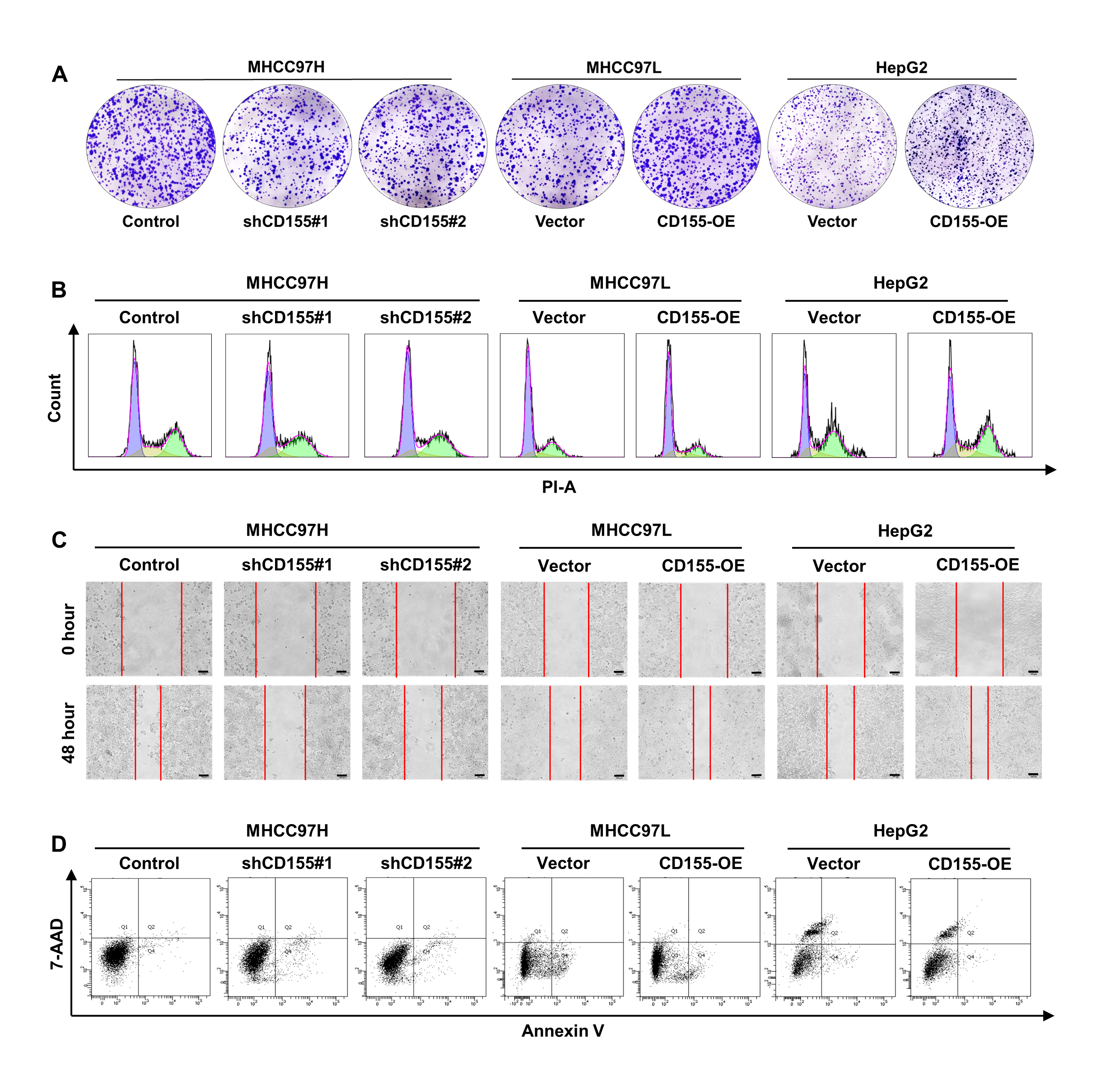

Supplement: Supplementary file 7 — Supporting Information [file CTM2-12-e794-s002.TIF]

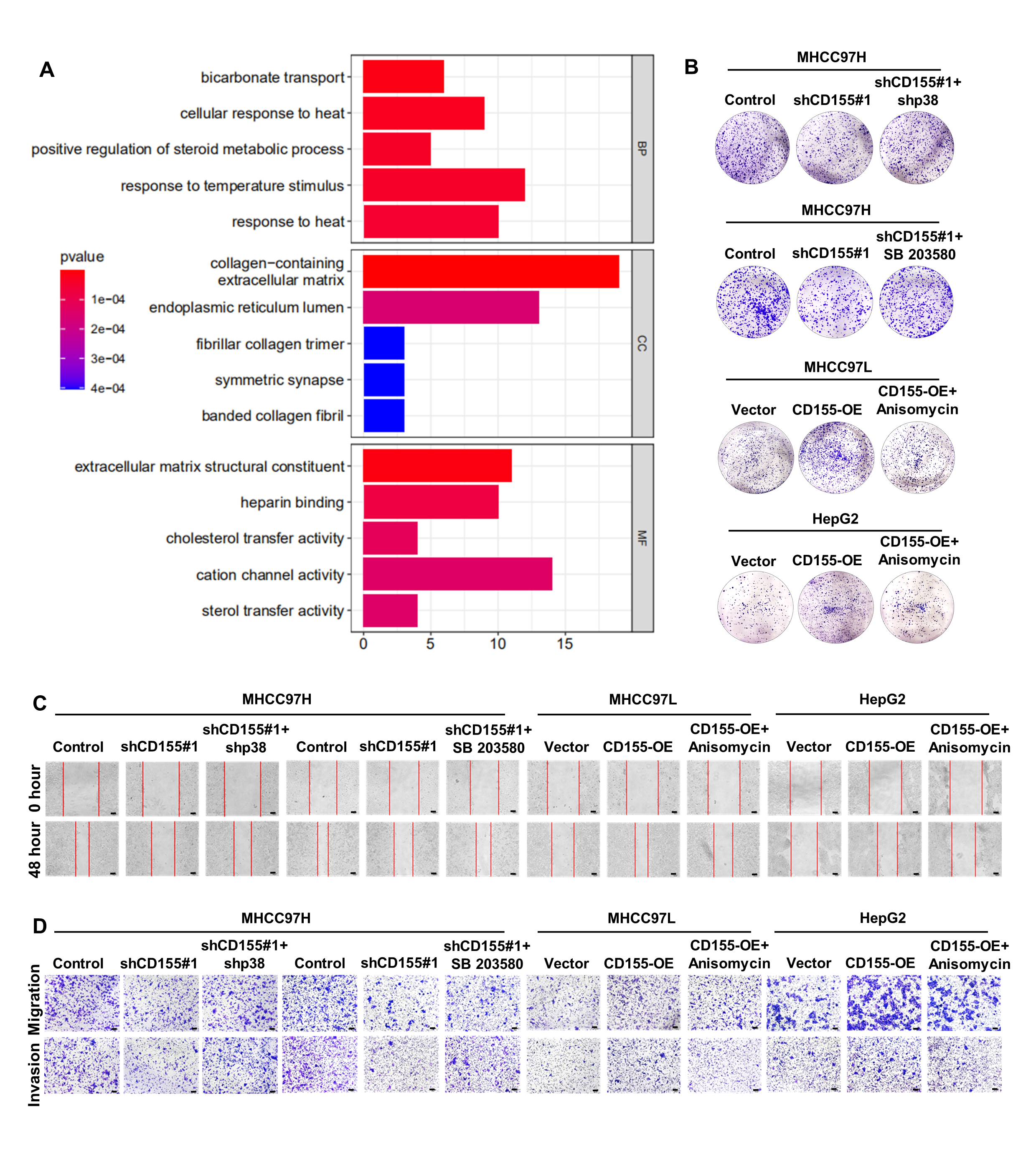

Supplement: Supplementary file 8 — Supporting Information [file CTM2-12-e794-s010.TIF]

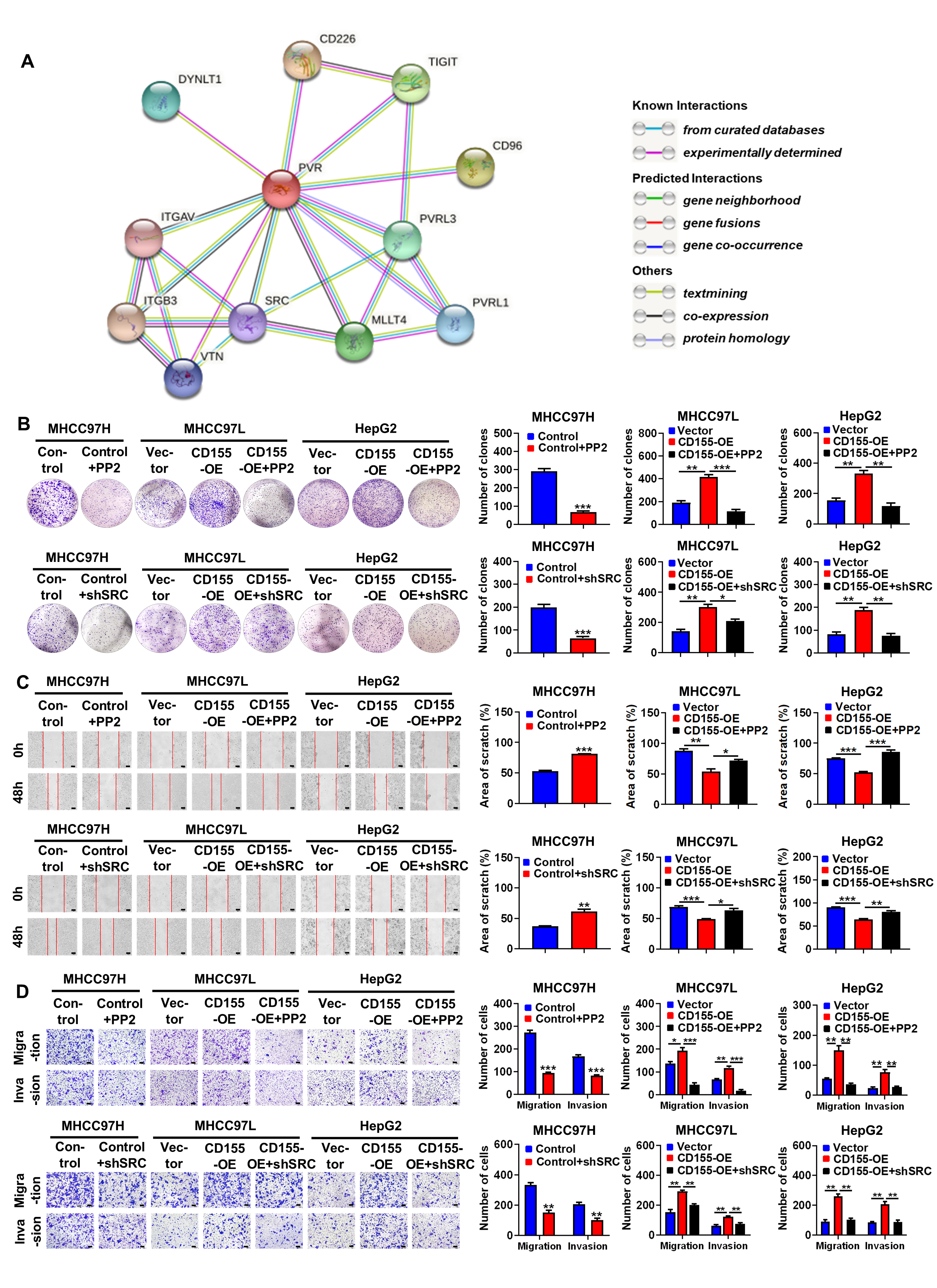

Supplement: Supplementary file 9 — Supporting Information [file CTM2-12-e794-s003.TIF]

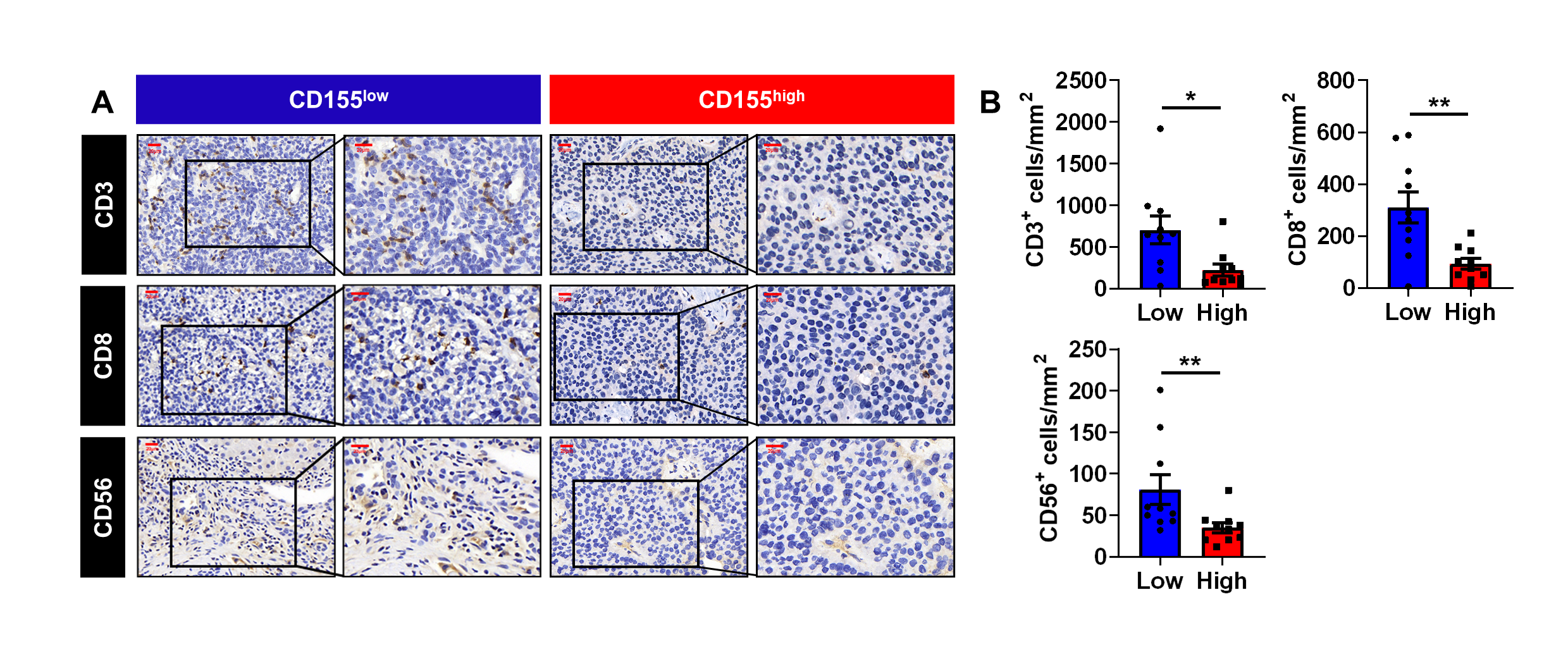

Supplement: Supplementary file 10 — Supporting Information [file CTM2-12-e794-s009.TIF]
